# Structure and dynamics of the membrane attaching nitric oxide transporter nitrophorin 7

**DOI:** 10.12688/f1000research.6060.1

**Published:** 2015-02-13

**Authors:** Markus Knipp, Hideaki Ogata, Giancarlo Soavi, Giulio Cerullo, Alessandro Allegri, Stefania Abbruzzetti, Stefano Bruno, Cristiano Viappiani, Axel Bidon-Chanal, F. Javier Luque

**Affiliations:** 1 Max-Planck-Institut für Chemische Energiekonversion, Mülheim an der Ruhr, 45470, Germany; 2 Dipartimento di Fisica e Scienze della Terra, Università di Parma, Parma, 43124, Italy; 3 Dipartimento di Bioscienze, Università di Parma, Parma, 43124, Italy; 4 NEST, Istituto Nanoscienze, Consiglio Nazionale delle Ricerche, Pisa, 56127, Italy; 5 Dipartimento di Farmacia, Università di Parma, Parma, 43124, Italy; 6 Dipartimento di Fisica, Politecnico di Milano, Milano, 20133, Italy; 7 Departament de Fisicoquímica, Facultat de Farmàcia and Institute of Biomedicine, Universitat de Barcelona, Santa Coloma de Gramenet, E-08921, Spain

**Keywords:** Nitrophorin 7, X-ray crystallography, ultrafast laser flash photolysis, ligand rebinding, molecular dynamics simulations, inner cavities, kinetics mechanism

## Abstract

Nitrophorins represent a unique class of heme proteins that are able to perform the delicate transportation and release of the free-radical gaseous messenger nitric oxide (NO) in a pH-triggered manner. Besides its ability to bind to phospholipid membranes, the N-terminus contains an additional Leu-Pro-Gly stretch, which is a unique sequence trait, and the heme cavity is significantly altered with respect to other nitrophorins. These distinctive features encouraged us to solve the X-ray crystallographic structures of NP7 at low and high pH and bound with different heme ligands (nitric oxide, histamine, imidazole). The overall fold of the lipocalin motif is well preserved in the different X-ray structures and resembles the fold of other nitrophorins. However, a chain-like arrangement in the crystal lattice due to a number of head-to-tail electrostatic stabilizing interactions is found in NP7. Furthermore, the X-ray structures also reveal ligand-dependent changes in the orientation of the heme, as well as in specific interactions between the A-B and G-H loops, which are considered to be relevant for the biological function of nitrophorins. Fast and ultrafast laser triggered ligand rebinding experiments demonstrate the pH-dependent ligand migration within the cavities and the exit route. Finally, the topological distribution of pockets located around the heme as well as from inner cavities present at the rear of the protein provides a distinctive feature in NP7, so that while a loop gated exit mechanism to the solvent has been proposed for most nitrophorins, a more complex mechanism that involves several interconnected gas hosting cavities is proposed for NP7.

## Introduction

Nitrophorins (NPs) comprise a family of heme binding proteins that originate from the saliva of the *Rhodnius* species of Triatomid bugs ^1, 2^. These insects use the heme iron as an anchor to store nitric oxide (NO), which is released during feeding on a host while the saliva is continuously pumped into the host tissue ^3^. NO assists the feeding process by inhibition of blood coagulation and increase of blood vessel diameter. The heme cofactor is located inside an antiparallel 8-stranded β-barrel, a typical motif of the lipocalin fold ^4^. The lipocalins represent a widespread family of single domain proteins that typically bind hydrophobic small molecules inside their β-barrel. Prominent examples of lipocalins are the retinol binding protein or the cow milk allergen β-lactoglobulin ^4, 5^. For a long time NPs were regarded as the only lipocalin able to bind heme. Recently, we reported on the human lipocalin α _1_-microglobulin (α _1_m) for which heme binding was previously suggested ^6^ and is now established ^7^. However, heme coordination is accomplished in a completely different manner in α _1_m, which forms an unusual [(α _1_m)(heme) _2_] _3_ complex of yet unknown structure.

At present, five NPs from the species *Rhodnius prolixus*, designated NP1, NP2, NP3, NP4 and NP7, have been recombinantly expressed and characterized in detail. High-resolution X-ray crystal structures were solved from NP1, NP2 and NP4. While NP1 and NP2 share very high amino acid sequence identity with NP4 (88%) and NP3 (81%), respectively, NP7 exhibits notable differences, which are mostly reflected in its high p *I* of 9.2 compared to the range of 6.1–6.5 covered by NP1-4 ^8^. This extreme divergence is a consequence of the presence of 27 Lys out of a total of 185 residues, which, as homology modeling has revealed, cluster mostly at the surface at the side opposite to the heme pocket ^8^. Thus, in contrast to the other NPs, NP7 binds to negatively charged phospholipid membranes with high affinity (4.8 nM) ^9, 10^.

Since the administration of NO as a pharmacologically active compound is of major interest for the treatment of cardiovascular diseases or cancer ^11^, the understanding of the structural and dynamic aspects of NO transport and storage by NPs is a major motivation for their detailed investigation. Here we report on seven crystal structures of NP7 at low and high pH and with different heme ligands. Moreover, molecular dynamics (MD) simulations were carried out to examine specific features of NP7, including the structural impact of the three extra residues present at the N-terminus of NP7, which represents a unique distinctive trait among NPs, and the topology of inner cavities. Finally, the structural studies were accompanied by fast and ultra-fast laser-flash photolysis experiments, which allowed to characterize the ligand migration and binding in NP7 from the 10 ^-12^ to the 10 ^-3^ s time-scale.

## Materials and methods

### Expression of NP7

The protein was expressed, reconstituted, and purified as was described previously ^10, 12^. The extended purification by cation exchange chromatography using a Ca ^2+^-charged chelating Sepharose (GE Healthcare) ^13^ was essential for the successful crystallization. Protein preparations were judged by SDS-PAGE to be >95% pure. MALDI TOF MS (Voyager DE Pro, Applied Biosystems) confirms the correct molecular masses including an initial Met-0 residue and accounting for two Cys–Cys disulfides (calculated for [NP7 + H] ^+^: 20,969 Da, observed: 20,966 ± 20 Da). Proteins were kept at –20ºC in 200 mM NaO *Ac*/HO *Ac* (Carl Roth), 10% (v/v) glycerol (Carl Roth) (pH 5.0) until use.

### X-ray crystallography

Protein crystals were obtained from 10 mg mL ^-1^ NP7 in 10 mM NaO *Ac*/HO *Ac* (pH 5.5) using the vapor-diffusion method ^12, 14^ upon mixing with an equal volume of crystallization solution (Hampton research) as indicated in Table 1. The crystals were soaked for 10 min on ice in the respective crystallization solution plus 15% glycerol as a cryo-protectant. Where needed, heme ligands were added into the cryo-protectant solution. Afterwards, the crystals were immediately frozen in liquid nitrogen and kept there until the measurement. Diffraction data sets were collected at 100 K using the beamlines, BL14.2 at BESSYII (Helmholtz-Zentrum Berlin, Germany) and PXII at SLS (Villigen, Switzerland). The data set was processed with XDS ^15^ and CCP4 ^16^. The molecular-replacement method was applied using M olR ep ^16^ and an initial model from NP4 (PDB code 3MVF) ^17^. Model building and refinement were carried out using W inC oot ^18^ and P henix ^19^, respectively. Data collection and refinement statistics are summarized in Table 1. PHENIX was used to check the stereochemical properties.

### Ultrafast spectroscopy

Samples for laser flash photolysis experiments were prepared by equilibrating the solutions in a sealed 0.2×1 cm-quartz cuvette connected to a tonometer with 0.1 or 1 atm CO. Na _2_S _2_O _4 _was then titrated anaerobically into the solution while formation of the CO adduct was monitored by absorption spectroscopy. The solubility of CO in water was such that its concentration was 1.05 mM at 10°C when the solution was equilibrated with 1 atm CO. The temperature dependence of CO solubility ^20^ was taken into account in the numerical analysis. The numerical analysis of the rebinding kinetics was described in detail previously ^21, 22^.

The experimental setup used for the pump-probe studies has been described ^23^. The sample was excited with a 70-fs,-10 nm bandwidth pump pulse centered at 530 nm obtained by a home-made optical parametric amplifier, driven by a regeneratively amplified Ti:sapphire laser at 1 kHz repetition rate. The probe pulse was a broadband single-filament white-light continuum generated in CaF _2_, covering a spectral range from 350 nm to 700 nm approximately. The pump pulse passed through a delay line and was then overlapped with the probe beam on the sample. The transmitted probe light was dispersed on an optical multichannel analyzer equipped with fast electronics, allowing single-shot recording of the probe spectrum at the full 1 kHz repetition rate. By changing the pump-probe delay we recorded 2D maps of the differential absorption (Δ *A*), as a function of probe wavelength and delay. The temporal resolution was determined to be approximately 150 fs and the sensitivity, for each probe wavelength, was better than 10 ^-4^.

### Nanosecond laser flash photolysis

The experimental setup was described in detail elsewhere ^21^. Photolysis of CO complexes was obtained using the second harmonic (532 nm) of a Q-switched Nd:YAG laser (Surelite-Continuum II-10) at 10 Hz repetition rate. We used a cw output of a 75 W Xe arc lamp as probe beam, a 5-stages photomultiplier (Applied Photophysics) for detection and a digital oscilloscope (LeCroy LT374, 500 MHz, 4 GS s ^−1^) for digitizing the voltage signal. A spectrograph (MS257 Lot-Oriel) was used to select the monitoring wavelength (436 nm) and to remove the stray light from the pump laser. The overall temporal resolution was determined to be about 10 ns and the sensitivity was better than 10 ^-4^. The sample holder was accurately temperature-controlled with a Peltier element, allowing a temperature stability of better than 0.1°C. Experiments were performed at 20°C.

### Data analysis

The analysis of the entire CO rebinding kinetics required the use of a detailed kinetic scheme (see section **Ligand rebinding kinetics** for details) in order to estimate the microscopic rate constants. The differential equations associated with the kinetic schemes were solved numerically, and the rate constants were optimized to describe simultaneously the experimental data at two different CO concentrations. Numerical solutions to set coupled differential equations associated with reaction schemes were determined by using the function ODE15s within Matlab 7.0 (The MathWorks, Inc., Natick, MA). Fitting of the numerical solution to experimental data was obtained with a Matlab version of the optimization package Minuit (CERN).

Spectra acquired in the fs-ps time scale were analyzed by singular value decomposition (SVD), performed using the software Matlab. Our data matrix ***D***, consists of differential absorption measured as a function of two variables: the wavelength of the probe beam and the time delay between the pump and the probe pulses. The singular value decomposition of ***D*** can be written as:

***D*** = ***USV*** ^T^        (1)

The meaning of the symbols is the following: columns of matrix ***U*** are a set of linearly independent, orthonormal basis spectra; columns of ***V*** represent the amplitudes of these basis spectra as a function of time; ***V*** ^T^ is the transpose of matrix ***V***; matrix ***S*** is a diagonal matrix of non-negative singular values that give the extent of the contributions of each of the products of the *i*-th column vectors *U _i_V _i_* ^T^ to the data matrix ***D***. The selected components can be further screened by evaluating the autocorrelation of the corresponding columns of ***U*** and ***V***, rejecting the component if the autocorrelation falls below 0.8 ^24^.

### MD simulations

MD simulations were used to explore the conformational space of wild type NP7 and its NP7(Δ1–3) variant, specifically regarding the structural flexibility of the N-terminus and the loops that shape the mouth of the heme cavity. The molecular systems were modeled from the X-ray structures of NO-bound NP4 at pH 5.6 and 7.4 (PDB ID: 1X8O, resolution of 1.0 Å; 1X8N; resolution of 1.1 Å) ^25^ as representative systems at low and high pH. Following previous studies ^26– 28^, standard ionization states were assigned to all the residues with the only exception of Asp32, which was protonated at low pH and ionized at high pH. The two native disulfide bridges were also imposed between residues Cys5 and Cys124 and between Cys42 and Cys173 ^29^. Finally, the heme was modeled in the **A** orientation, which was shown to be the thermodynamically favored orientation for both NP7 and Met-NP7(Δ1–3) ^8, 30^.

Simulations were run using the charmm22 ^31^ force field and the NAMD program ^32^. The protein was immersed in a pre-equilibrated cubic box (~70 Å per side) of TIP3P ^33^ water molecules. Bonds involving hydrogen atoms were constrained at their equilibrium length using SHAKE and SETTLE algorithms, in conjunction with a 2 fs time step for the integration of the Newton's equations. Trajectories were collected in the NPT (1 atm, 300 K) ensemble using periodic boundary conditions and Ewald sums (grid spacing of 1 Å) for long-range electrostatic interactions. A multistep protocol was used to minimize and equilibrate the system. Thus, the energy minimization was first performed for the hydrogen atoms, then water molecules, and finally the whole system. The equilibration was performed by heating from 100 to 298 K in four 200-ps steps at constant volumen. Then, a 200 ps MD at constant pressure and temperature was run. The final structure was used for the production MD runs, which covered 100 ns. Frames were collected at 1 ps intervals, which were subsequently used to analyze the trajectories.

## Results and discussion

### X-Ray crystallography

Conditions for the crystallization of NP7 at pH 7.8 were previously reported ^12^. The addition of di- and polyanionic substances to compensate for the positively charged surface of NP7 that is responsible for the binding to negatively charged phospholipid membranes was crucial for the crystal formation ^9, 10^. The previously reported crystals diffracted to a resolution of 1.8 Å ^12^. Further optimization of the crystallization conditions resulted in crystals that diffract to even higher resolution down to 1.29 Å ( Table 1). It was also possible to crystallize the protein at low pH conditions, i.e., pH 5.8. Since charge compensation for the Lys surface patch was crucial, we also tried to add the amine coordinating bisphosphonate (“lysine tweezer”) **1** ( Scheme 1), which is known to be a Lys-specific binder ^34^, resulting in high resolution crystals. Another additive that turned out to be successful was the Gly–Gly–Gly tripeptide.

**Figure:**
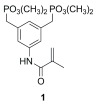
Scheme 1.

**Table 1.** Refinement statistics of the crystal structures of NP7 obtained with various heme ligands and under various crystallization conditions. The highest resolution shell is shown in parenthesis.

heme ligand	H _2_O ^a^	H _2_O ^b^	NO ^c^	Hm ^c^	ImH ^d^	Gly–Gly–Gly ^c^
pH	5.8	7.8	7.8	7.8	7.8	7.8
PDB code	4XMC	4XMD	4XME	4XMF	4XMG	4XMH
***Data collection***					
X-ray source	SLS PXII	BESSYII BL14.2	SLS PXII	BESSYII BL14.2	SLS PXII	SLS PXII
Wavelength (Å)	0.99999	0.91841	0.99999	0.91841	0.800010	0.99998
Space group	*P*2 _1_	*P*2 _1_	*P*2 _1_	*P*2 _1_	*P*2 _1_	*P*2 _1_
Unit-cell parameters *a* (Å) *b* (Å) *c* (Å) *β* (°)	38.42, 67.00, 39.00 116.6	38.31, 66.81, 39.03 116.9	38.89 67.05 39.08 117.3	38.34 66.72 38.83 116.9	38.26, 66.75, 38.56 116.5	38.37 66.93 38.89 116.8
Resolution (Å)	34.88-1.42 (1.46-1.42)	30.42-1.60 (1.64-1.60)	34.71-1.29 (1.32-1.29)	30.74-1.60 (1.64-1.60)	34.25-1.80 (1.85-1.80)	34.71-1.29 (1.37-1.29)
No. of observed reflections	106086	86820	140029	86063	52612	126311
No. of unique reflections	59987	23143	83829	22417	15836	41095
*R*_merge_	0.033 (0.646)	0.040 (0.486)	0.032 (0.925)	0.067 (0.780)	0.083 (0.439)	0.024 (0.290)
Completeness (%)	91.3 (71.4)	99.4 (99.8)	94.0 (84.0)	96.9 (95.5)	97.6 (96.8)	92.8 (64.7)
< *I/σ*( *I*)>	11.0 (1.4)	20.5 (3.0)	9.5 (1.0)	15.3 (1.9)	9.2 (3.0)	21.4 (2.3)
***Refinement***					
Resolution	34.9-1.4	30.4-1.6	34.7-1.3	30.7-1.6	34.3-1.8	34.7-1.3
*R* (%)	16.1	13.5	17.1	15.8	17.8	15.5
*R*_free_ (%)	19.4	19.2	20.5	20.7	21.7	18.2
No. of residues	184	184	184	184	184	185
No. of water	80	199	150	126	120	162
Rmsd bond length (Å)	0.006	0.006	0.010	0.007	0.008	0.006
Rmsd bond angle (°)	1.042	1.026	1.074	1.024	1.157	1.067
Ramachandran plot Outliers (%) Favored (%)	0.0 99.45	0.0 98.36	0.0 99.45	0.0 99.45	0.0 98.91	0.0 98.92
Average B Protein (Å ^2^) Ligand heme (Å ^2^) Ligand (Å ^2^) Solvent (Å ^2^)	28.6 29.5 27.2 35.6	27.2 34.2 52.7 40.5	22.8 20.0 17.3 30.0	21.9 27.4 30.3 29.4	26.4 23.9 21.9 29.6	22.1 19.1 27.7 30.5
Heme orientation	B	B	B	B	A	B

The crystallization conditions (all reagents from Hampton Research) were; a) 25%(w/v) PEG 3350, 0.1M MES monohydrate pH 5.8, 0.25%(w/v) Gly-Gly, 0.25%(w/v) Gly-Gly-Gly, 0.25%(w/v) Gly-Gly-Gly-Gly, 0.25%(w/v) pentaglycine, 0.02 M HEPES sodium pH 6.8. b) 25%(w/v) PEG 3350, 0.1 M bis-Tris propane pH 7.8, 0.02 M HEPES pH 6.8, 0.25%(w/v) naphthalene-1,3,6-trisulfonic acid trisodium salt hydrate, 0.25%(w/v) 2,6-naphthalenedisulfonic acid disodium salt, 0.25%(w/v) 4-aminobenzoic acid, 0.25%(w/v) 5-sulfosalicylic acid dehydrate. c) 25%(w/v) PEG 3350, 0.1 M bis-Tris propane pH 7.8, 0.25%(w/v) Gly-Gly, 0.25%(w/v) Gly-Gly-Gly, 0.25%(w/v) Gly-Gly-Gly-Gly, 0.25%(w/v) pentaglycine, 0.02 M HEPES sodium pH 6.8. d) 25%(w/v) PEG 3350, 0.1 M bis-Tris propane pH 7.8. 0.25%(w/v) hexamminecobalt(III) chloride, 0.25%(w/v) salicylamide, 0.25%(w/v) sulfanilamide, 0.25%(w/v) vanilic acid, 0.02 M HEPES sodium pH 6.8.

To obtain different iron-liganded structures, the crystals were soaked with imidazole (ImH), histamine (Hm), or the NO donating compound DEA/NONOate for 10 min at room temperature. Upon incubation with the mother liquor containing 15% of glycerol as a cryo-protectant, crystals were frozen in liquid nitrogen. Diffraction experiments were carried out at two different synchrotrons at 100 K.

All crystals occupied the space group *P*2 _1_. Refinement of the crystal structures was obtained through the molecular replacement method using the structure of NP4 (PDB code 3MVF) ^17^ as a template. The refinement statistics are summarized in Table 1. It should be mentioned that no electron density of the additives (see Table 1 footnotes) except for the Gly–Gly–Gly tripeptide was observed.

### Protein fold

**Figure 1.**
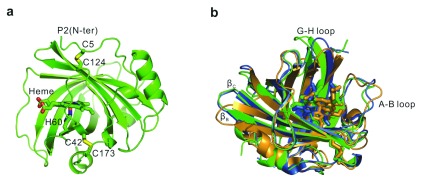
Representation of the backbone structure of X-ray NP7 structures. ( **a**) Overall fold of NP7. ( **b**) Overall fold of NP7 (green) in comparison to NP2 (blue) (PDB code, 2A3F) and NP4 (orange) (PDB code, 1YWD).

The overall fold of NP7 resembles that of other NPs. Eight anti-parallel β-strands (A to H) form a barrel that hosts the heme cofactor including the ligation by the proximal His60 residue. The structural identity reflected by the RMSD values obtained from the comparison of the backbone atoms of two of the isoforms correlates with the amino acid sequence identities ( Table 2). Figure 1 displays the overall fold compared to those of NP2 and NP4. The core of the lipocalin fold is well superposed in all cases. The position of the two disulfide bridges, which is a common trait of the NPs, is very similar among all the NPs. The largest differences are found in the A-B, B-C and G-H loops. In detail, the bending of the β-strands (β _B_ and β _C_) is significantly more similar in the case of NP7 and NP2 compared to NP4. This is also true for the AB-loop. On the other hand, significant differences are found in the spatial arrangement of the G-H loop, which is markedly bent in NP7 compared to NP2 and NP4 ( Figure 1).

**Table 2.** Comparison of the RMSD values (Å; upper triangle) and amino acid sequence identities (%; lower triangle) of NP1, 2, 4, and 7 ^*a*^.

	NP1	NP2	NP4	NP7
**NP1**		1.46	0.79	1.54
**NP2**	45		1.51	1.26
**NP4**	88	44		1.63
**NP7**	40	60	41	

^*a*^ The RMSD calculation is based on the following PDB files: NP1, 2NP1; NP2, 2A3F; NP4, 1YWD; NP7, 4XMC.

### Protein surface

**Figure 2.**
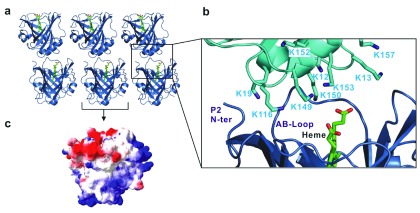
Head-to-tail arrangement of NP7 in the crystal lattice. ( **a**) Arrangement of molecules in the crystal lattice of NP7. ( **b**) Crystal contact between two neighboring molecules in the NP7 crystal. ( **c**) The electrostatic potential of NP7. The red and blue colors show the negative and positive potentials, respectively.

**Figure S1.**
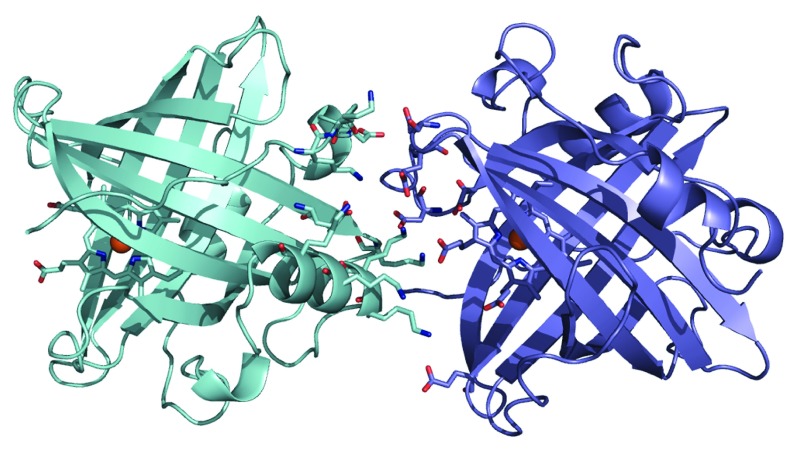
Distribution of positively and negatively charged residues in the head-to-tail interface between NP7 molecules.

One of the most interesting features of NP7 is its ability to bind to negatively charged membranes. The crystal structures demonstrate the extensive clustering of Lys side-chains at the protein surface opposite the heme pocket that accomplishes the strong protein-membrane interaction. On the other hand, the side of the heme mouth has a negative charge potential leading to a total bipolar charge distribution. It was previously noticed that NP7 tends to aggregate at elevated concentrations ^8, 35^, which can be explained by a charge stabilized aggregation process. X-ray crystallography indeed supports such a head-to-tail interaction, which involves a variety of salt bridges ( Figure S1), leading to chain-like arrangement in the crystal lattice ( Figure 2a).

### Heme pocket

**Figure 3.**
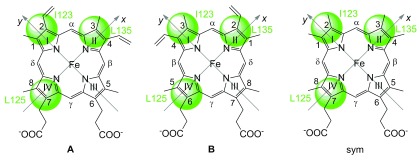
Heme *b* nomenclature and heme orientation inside the pocket of NP7. The heme pocket of NP7 is indicated by the three green circles corresponding to the position of the three aliphatic side-chains pointing from the top of the distal pocket onto the heme plane. The two possible heme orientations are indicated as **A** and **B**.

The presence of Glu27 residue in the heme pocket of NP7 is unique among NPs ^36, 37^. Upon homology modeling it was noticed that the Glu27 carboxylate must somewhat interfere with the hydrophobic site of the cofactor, i.e., its vinyl and methyl substituents. Only **A** orientation is observed in NP7 ^8, 30^, whereas **B** orientation is favored in NP2 ^38, 39^ ( Figure 3). Interestingly, the mutant that converts Glu27 to Val, i.e. the residue found in all the other NPs (NP7(E27V)), demonstrated that the heme orientation was reversed from **A** to **B**, whereas the replacement of Glu27 by Gln, NP7(E27Q), did not change the heme orientation compared to the wild type, and replacement of Val24 by Glu in NP2, NP2(V24E), resulted in the **B** → **A** reorientation of the cofactor ^30^.

**Figure 4.**
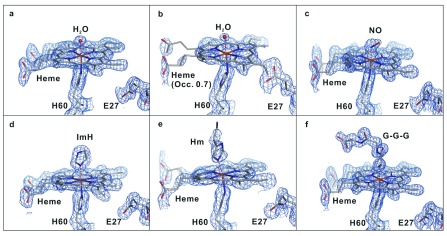
Electron density maps in ligand-bound NP7. 2Fo-Fc electron density map (contoured at 1σ) of the heme pocket of ( **a**) NP7 at pH 5.8, ( **b**) NP7 at pH 7.8, ( **c**) NP7[NO], ( **d**) NP7[ImH], ( **e**) NP7[Hm], and ( **f**) NP7[Gly-Gly-Gly].

In the crystal structure of NP7[ImH] the electron density map clearly reflects the presence of heme **A** orientation, as expected for NP7 (see above). However, to our surprise, the NP7 structure that crystallized with Gly–Gly–Gly has heme **B** orientation ( Table 1 and Figure 4). Thus, it may be concluded that the insertion of this ligand leads to a widening of the heme pocket, which may allow the heme to turn. Since the structures of NP7[NO], NP7[Hm] and unliganded NP7 at pH 5.8 were derived from crystal soaking and displacement of the Gly–Gly–Gly ligand, the heme consequently was found in the heme **B** orientation as the crystal lattice does not allow the cofactor to turn. However for NP7[NO], **A** orientation was demonstrated by circular dichroism spectroscopy ^30^. Caution is needed regarding the presence of **B** orientation in unliganded NP7 at pH 7.8, since it could not be properly assigned due to the loosely occupied heme ( Table 1).

In the electron density map derived from the diffraction pattern of the unliganded NP7 at pH 7.8 the heme was very poorly defined, while the rest of the structure was very well defined ( Figure 4b). In contrast, the electron density of the cofactor bound to ImH, Hm and NO was well defined ( Figures 4c–e). Since the ferriheme iron undergoes rapid photo reduction during the recording of diffraction patterns ^40– 43^, the crystal structures display the Fe ^II^ state. We had previously reported that the Fe ^II^–N _His60_ bond in NP7 is surprisingly weak, so that the reduction of NP7 leads to the equilibrium noted in Equation 2 at neutral pH (p *K*_a_ = 7.8) ^44^.

H _2_O + Fe ^II^(ppIX)–N _His60_ ⇆ H _2_O–Fe ^II^(ppIX) + N _His60_      (2)

Overall, these findings suggest that the ill-defined heme density of the unliganded NP7 reflects the movement of the cofactor inside the heme pocket according to the two coordination states. The binding of the π donors ImH, Hm or CO has a positive *trans* effect, which stabilizes the Fe ^II^–N _His60_ bond, so that the heme resides in the pocket. Interestingly, NO has a negative *trans* effect due to the overlap of its anti-bonding π* orbital with the iron d _*z*²_ orbital, thus weakening the Fe–N _His_ σ bond ^45– 47^.

**Figure 5.**
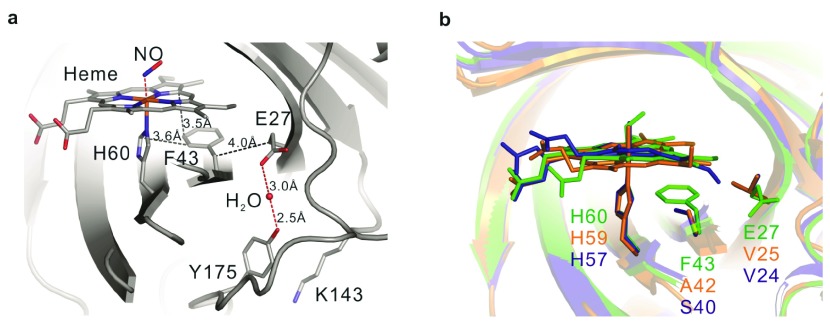
Local structural details of Glu27. Spatial location relative ( **a**) to the heme cofactor and ( **b**) with respect to His60 and Phe43. For comparison, the structures of NP7(green), NP2 (blue) and NP4 (orange) are displayed.

The crystal structures allow detailed examination of Glu27 ( Figure 5a). The side-chain is folded away from the hydrophobic heme side toward the interior of the structure, where it is involved in a network of H-bonding contacts. This includes a single water that is further coordinated to Tyr175 near the protein surface. It was previously found that the mutation Glu27→Gln has a remarkable destabilizing effect on the NP7 fold ^30^. It can now be understood that the negative charge of Glu27 attracts the Tyr175 hydroxyl group, which forms an important interaction. On the other hand, the dense packing of side-chains next to His60 was identified as another destabilizing factor of the Fe ^II^–N _His60_ bond, where Phe43 plays a crucial role ^44^. Figure 5b shows the arrangement in comparison to the crystal structures of NP2 and NP4. Phe43 is oriented parallel to the heme plane with a distance of 3.5 Ǻ leading to π-stacking between the two aromatic rings. Moreover, the phenyl ring is perpendicularly oriented toward the His60 plane with a distance of 3.6 Ǻ. The distance between Glu27:C _β_ and Phe43:C _β_ is 4.0 Ǻ, while Glu27 also H-bonds to Phe43:NH.

### Binding of NO

In the crystal structure, NO is bound with ∠(Fe–N–O) = 124°, which corresponds to a reduced iron, i.e. Fe ^II^(NO). In the case of NP1[NO] a similar angle of 123° to 135° was reported ^48^. For NP4[NO] two orientations of 110° and 177° were assumed because the electron density could not be sufficiently fit with a single conformation ^49^. The data were interpreted by the parallel co-existence of photoreduced Fe ^II^(NO) and some residual Fe ^III^(NO) in the frozen crystal. However, according to our ^41^ and other ^40, 43^ work, photoreduction occurs even in frozen crystals within the first seconds of data collection, thus the presence of significant amounts of NP4[Fe ^III^(NO)] seems very unlikely. Also in the case of NP7[NO] some of the electron density could not be sufficiently fit with the presence of a single NO configuration ( Figure 4c). However, this may reflect an incomplete occupancy of the axial Fe site with NO and displacement with water since the protein crystals were prepared and transported in the Fe ^III^(NO) form to the synchrotron, whereby loss of NO gas is possible.

**Figure 6.**
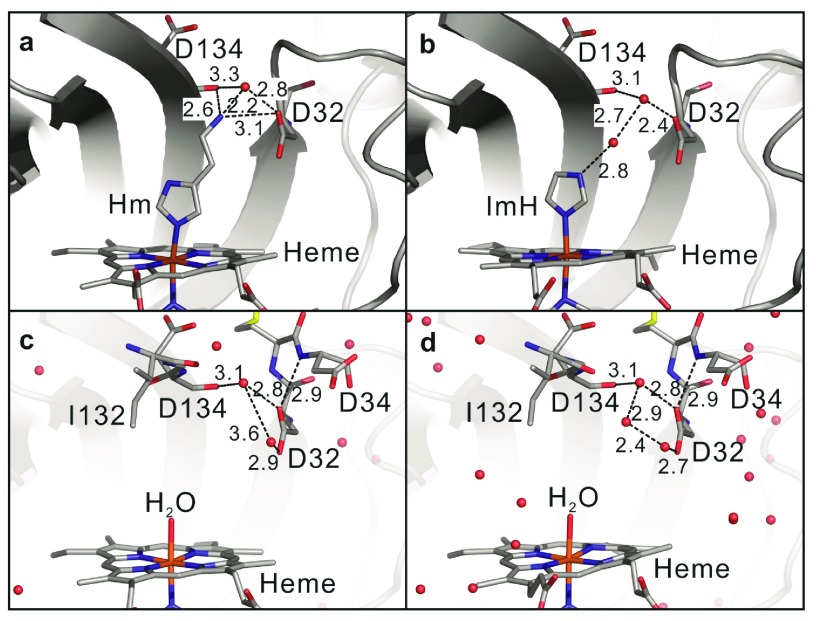
Local structural details of the heme pocket. ( **a**) Hm, ( **b**) ImH, ( **c**) pH 5.8 and ( **d**) pH 7.8. The numbers represent the bond distances (Å).

***Binding of imidazole and histamine.*** Similar to other NPs, the binding of Hm is accomplished not only through the coordination of heme iron, but also through the salt bridge of its ethylamine group with the Asp32 carboxylate ( Figure 6a). When ImH is bound, the missing ethylamine is compensated by H-bonding to a water molecule, which is then coordinated by Asp32 ( Figure 6b). However, in marked contrast to other NPs, the affinity constant for ImH and Hm was markedly decreased. While for NP2 the equilibrium constant ( *K*_eq_) was found to be 2.5 × 10 ^7^ M ^-1^ ^50^, for NP7 *K*_eq_ was 1.0 × 10 ^6^ M ^-1^ ^8^. This difference is even more pronounced for Hm, as the equilibrium constants are 1.0 × 10 ^8^ M ^-1^ ^50^ and 1.0 × 10 ^5^ M ^-1^ for NP2 and NP7 [8], respectively. The remarkable difference observed between the two ligands in NP7 as well as between NP2[Hm] and NP7[Hm] is not explained by the structures. However, taking into account that the N-terminus is expected to move into the space between the A-B and G-H loops, the disruption of the Asp32-Hm interaction is feasible. This is further supported by the finding that deletion of the N-terminal residues increases the affinity for Hm ( *K*_eq_(NP7(Δ1-3)) = 1.3 × 10 ^7^ M ^-1^). This trend is also found in the enhanced affinity for ImH ( *K*_eq_(NP7(Δ1-3)) = 3.2 × 10 ^7^ M ^-1^) ^8^, which compares with the value reported for NP2 (see above).

### pH dependence and N-terminus

It was previously suggested that the pH can have significant influence on the A-B and G-H loop conformations in NP4 due to the change in the protonation state of Asp30 (part of the A-B loop) ^25, 27, 51– 54^. While in the protonated state Asp30 binds to the backbone C=O of Leu130 (part of the G-H loop), it is detached upon deprotonation leading to the opening of the heme pocket ^51^. Based on this mechanism, a model for the pH-dependent NO release under biological conditions was developed. However, very limited pH dependences of the ligand release rates and equilibrium constants leave considerable doubts about this likely oversimplified model ^55^.

**Figure S2.**
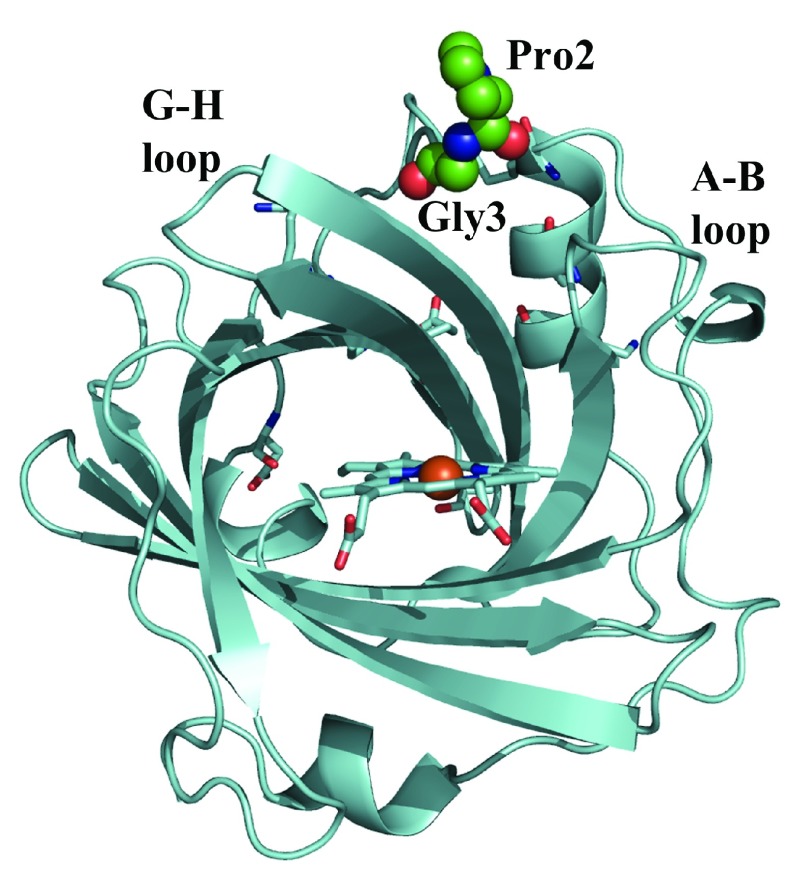
Representation of the backbone in the X-ray structure of NP7 solved at low pH (5.8). Residues Pro and Gly of the extra N-terminus stretch (Leu-Pro-Gly) found in NP7 are shown as green-coloured spheres. The spatial position of Leu, and the additional Met0 residue originating from the start codon of the expression system, were not seen in the X-ray crystal.

In contrast to NP1-4, where the pH dependence of the equilibrium constant is less than an order of magnitude, the difference for NP7 spans three orders of magnitude ^8^. However, no significant conformational differences were found between the X-ray structures collected at two different pHs (5.8 and 7.8; RMSD = 0.22 Å), even though this might simply arise from the packing of NP7 molecules in the crystal. On the other hand, in all NP7 structures Asp32 is bound to a water that is held by the backbone NH and C=O of Asp134, that is, the Asp32-Ile132 H-bond that corresponds to the Asp30-Leu130 H-bond in NP4 is not observed ( Figures 6c and 6d). Thus, there are significant differences in the key structural features during the transition between closed and open forms of the protein. However, it is unclear whether these differences are due to the head-to-tail arrangement (see above and Figure 2), or alternatively might be influenced by the distinct N-terminus stretch of NP7, which fills the region between A-B and G-H loops ( Figure S2).

**Figure S3.**
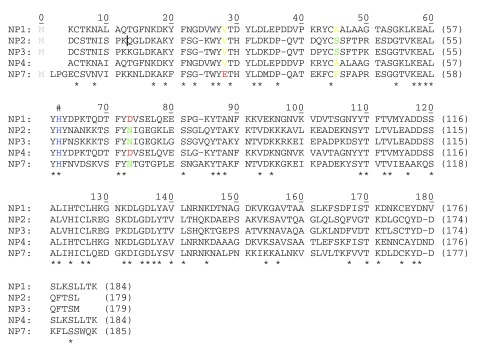
Amino acid sequence alignment of *R. prolixus* NP1 (Swiss-Port entry Q26239), NP2 (Swiss-Port entry Q26241), NP3 (Swiss-Port entry Q94733), NP4 (Swiss-Port entry Q94734), and NP7 (Swiss-Prot entry Q6PQK2) *(1)*. The total sequence identity among all five protein sequences amount to 33% (indicated by ‘*’). The proximal His is indicated by ‘#’. An initial Met residue (in grey) results in case of the recombinantly expressed proteins of NP1, NP2, NP3, and NP7 is not present in the mature proteins *in vivo* ( *2, 3*). Residues of relevance to this study are highlighted in color.

Upon maturation, a major difference of NP7 compared to all other NPs is its extended N-terminus ( Figure S3), which is characterized by the extension of the tripeptide Leu1–Pro2–Gly3 ^8, 56^. It should be mentioned that the N-terminus of the recombinant NP7 contains an additional Met0 residue originating from the start codon of the expression system. In the X-ray structures, the residues Met0 and Leu1 were not seen. As noted above, the crystal lattice involves a crystal contact between the A-B and G-H loops of one molecule and the positive patch of the back side of another molecule ( Figure 2b), where the sharp E-F loop Lys116 plays a major role, contributing to the head-to-tail arrangement via salt bridges with residues Asp34 and Asp32 (distances of 3.4 and 5.0 Å, respectively). As a consequence, the N-terminus is rather floppy sitting on top of the structure ( Figure S3).

A series of MD simulations were run to examine the conformational flexibility and potential interactions formed by the N-terminus stretch of NP7 in aqueous solution. To this end, MD simulations were carried out for models of the wild type protein representative of the closed and open states, and the results were compared with those obtained for the Δ(1-3) variant to ascertain the structural impact of the three extra residues. Furthermore, in order to avoid an artifactual bias due to packing effects, models of wild type and Δ(1-3) NP7 proteins were built up using the X-ray crystallographic structures of NO bound NP4 at pH 5.6 and 7.4 ^25^, because the conformations of the AB and GH loops are characteristic of the closed and open states, respectively ^52^. All the simulations led to stable trajectories, as noted in RMSD values comprised between 0.9 and 1.3 Å (data not shown).

**Figure 7.**
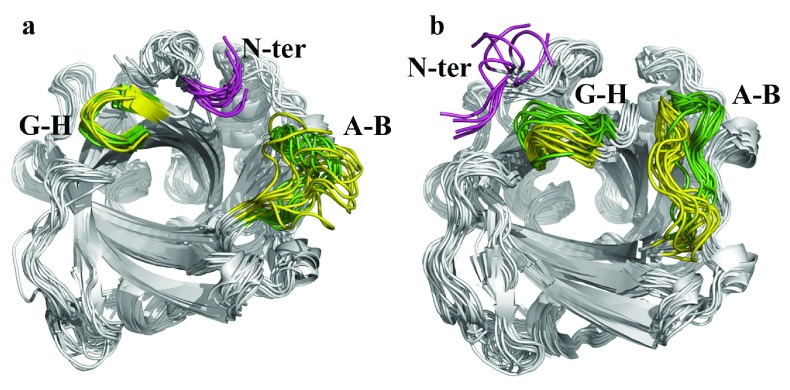
Structural analysis of MD sampled snapshots. Superposition of the snapshots taken every 10 ns from the MD trajectories sampled for the ( **a**) open and ( **b**) closed states of wild type NP7 and its Δ(1-3) variant. The backbone of the extra Leu-Pro-Gly stretch found in NP7 is shown in magenta, and the backbone of the A-B and G-H loops for wild type and Δ(1-3) NP7 is shown in green and yellow, respectively.

**Figure S4.**
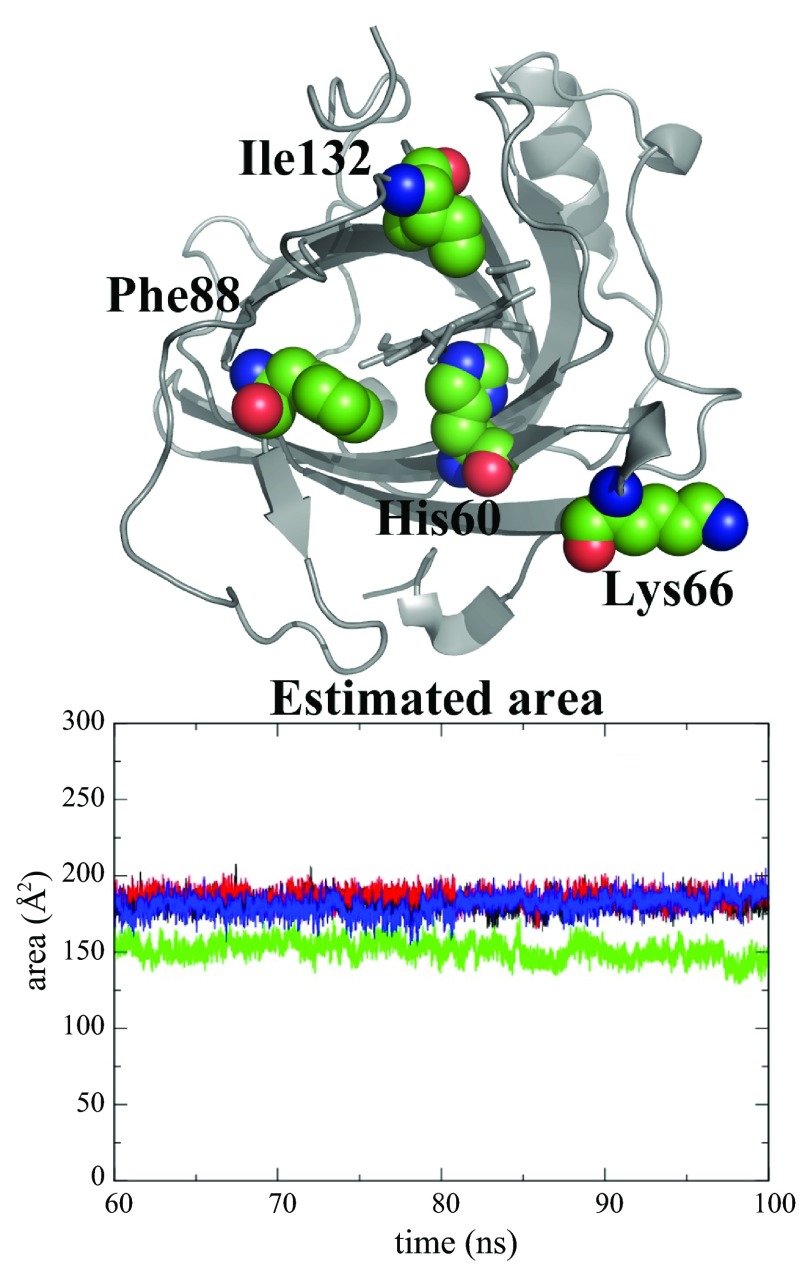
Time (ns) evolution of the estimated area (Å ^2^) of the heme cavity mouth determined from the triangles defined by the Cα atoms of residues His60, Lys66, Phe88 and Ile132. Wild type NP7 in open and closed states is shown as red and black lines; Δ(1-3) variant in open and closed states is shown as blue and green lines.

The behavior observed for the open forms of wild type NP7 and its Δ(1-3) variant is highly similar, mainly reflecting significant fluctuations of the A-B loop irrespective of the length of the N-terminus ( Figure 7a), which for NP7 fills the region between A-B and G-H loops, thus resembling the arrangement found in the X-ray structures of NP7 ( Figure S2). This structural resemblance is reduced in the closed state, because the A-B and G-H loops exhibit larger fluctuations in the wild type protein compared to the Δ(1-3) species ( Figure 7b), and this effect is accompanied by the enhanced conformational flexibility of the Leu1-Pro2-Gly3 stretch in NP7, which is in contrast with the more rigid structure adopted by the N-terminus in the Δ(1-3) variant. Interestingly, superposition of the snapshots sampled for wild type and Δ(1-3) proteins reveals a widening of the heme cavity mouth in the wild type protein ( Figure 7b), which would suggest a higher probability for the formation of transient pathways that connect the heme cavity with the bulk solvent. This is noted, for instance, in the larger area estimated for the heme cavity mouth for the closed states of the wild type NP7 and its Δ(1-3) variant ( Figure S4).

Overall, these findings could explain why deletion of the N-terminal residues increases the affinity for ImH and Hm, an effect that can presumably arise from the more compact nature of the heme cavity and lower fluctuations of the A-B and G-H loops in the Δ(1-3) protein. Finally, the enhanced flexibility of the closed form of wild type NP7 may also explain the larger sensitivity to pH dependence, since the access of water molecules to the heme cavity could facilitate breaking of the H-bond between Asp32 and Ile132, thus favoring deprotonation of Asp32 and hence the transition to the open state.

### Time resolved differential absorption spectra

**Figure S5.**
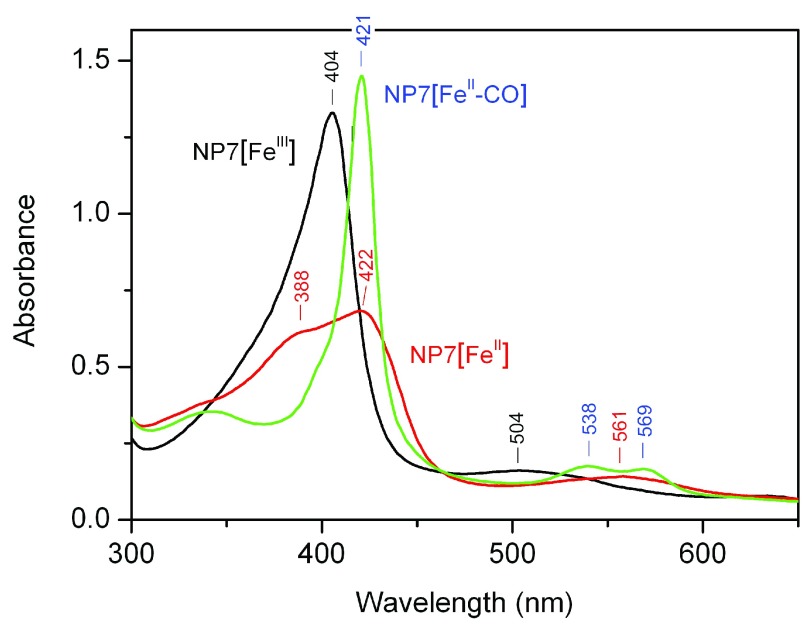
Absorption spectra of ferric and ferrous NP7 at pH 7.5. *Black*: NP7[Fe ^III^]; *red*: NP7[Fe ^II^]; *blue*: NP7[Fe ^II^–CO].

The availability of the NP7 structures now allows the combination with the studies of fast dynamics of the ligand-protein interaction. The Fe ^II^–CO complex was studied as a model for the isoelectronic Fe ^II^–NO because of its slower geminate rebinding rates. The change in absorption spectra of CO bound and unbound heme proteins ( Figure S5) allows the determination of time resolved differential absorption spectra after photolysis of the Fe–CO bond. In this study, NP7–CO was pumped with 70-fs laser pulses at 532 nm. Previous studies were performed using nanosecond laser flash photolysis, but the geminate rebinding was not resolved ^57^. We report here that geminate recombination starts in the picosecond range, and merging the subnanosecond kinetics with the nanosecond laser photolysis data allow us to obtain a full time course for ligand rebinding.

**Figure 8.**
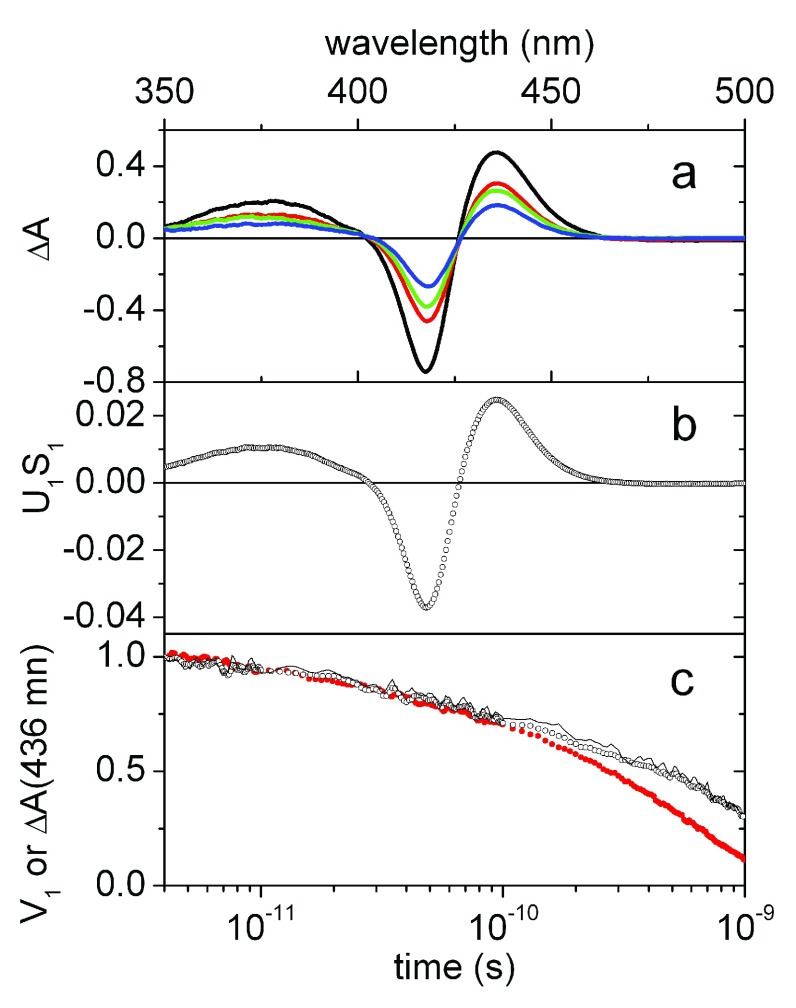
Spectral analysis. ( **a**) Time resolved differential absorption spectra for NP7–CO (pH 7.5, *T* = 20°C) following femtosecond photoexcitation at 532 nm at 4 ps (black line), 200 ps (red line), 400 ps (green line) and 800 ps (blue line) delay times. ( **b**) First spectral component ( *U*_1_) obtained from the SVD analysis of the time resolved differential absorption spectra multiplied by the corresponding singular value ( *S*_1_ = 0.23). ( **c**) Comparison between the time course of the amplitude *V*_1_ (open circles) of the main spectral component obtained from SVD, and the normalized transient absorbance at 436 nm (solid line) as a function of the delay time. The red filled circles report the amplitude *V*_1_ of the main spectral component obtained from the experiment conducted at pH 5.5.

Figure 8a shows time resolved differential absorption spectra measured after photolysis of the CO adduct of NP7 at selected time delays. Transient spectra correspond to the difference between the ground state absorption spectra of NP7[Fe ^II^–CO] and NP7[Fe ^II^], with clean isosbestic points at 402 nm and 426 nm. Accordingly, only one significant spectral component is retrieved from the SVD analysis of the spectra collected between 4 ps and 1 ns ( Figure 8b). As a consequence, the time course of the corresponding amplitude *V*_1_ perfectly matches the kinetics measured at 436 nm, but with significant noise reduction, as shown in Figure 8c. Similar results are obtained when the experiment is conducted at pH 5.5, where the time resolved differential absorption spectra have the same shape as those measured at pH 7.5. Also at this pH, only one spectral component is obtained from SVD and the time course of the amplitude is shown as red solid circles in Figure 8c. It is easily observed that the time course of *V*_1_ at acidic pH occurs with a higher rate, leading to a larger fraction of rebinding at the subnanosecond time scale.

### Ligand rebinding kinetics

We have previously reported the CO rebinding kinetics to NP7 after nanosecond laser photolysis, showing that the binding reaction can be followed by the absorbance change at 436 nm ^57^. In this work we now aim at reconstructing the full time course of CO rebinding, from a few picoseconds to reaction completion, occurring on the millisecond time scale. We have accomplished this by merging the CO rebinding kinetics determined in the femtosecond pump-probe and in the nanosecond laser flash photolysis experiments.

Due to inherent limitations of the methods employed to collect the kinetics in the two time regimes, the kinetics in the time interval between ~2 ns (the longest available delay in the pump-probe experiment) and 20 ns (the shortest time accessible without distortion due to the instrumental response function of the nanosecond laser flash photolysis setup) is not available. Thus, a connection of the two data sets is not straightforward. In order to obtain a single kinetic trace, we have first normalized the amplitude *V*_1_ retrieved from SVD analysis of the pump and probe experiment, taking advantage of the fact that the fraction of photoproduct with respect to that initially generated at time *t*_0_, i.e., *N*( *t*) = Δ *A*( *t*)/Δ *A*( *t*_0_), (with *t*> *t*_0_), decreases with time from unity. Unfortunately, it is not possible to extend this procedure to the nanosecond photolysis data since multiple photolysis events occur during the laser pulse under our experimental conditions, thus impairing a correct determination of the fraction of unliganded species surviving at 20 ns. We have therefore estimated the concentration of the unliganded molecules at the end of the temporal window of pump and probe experiments by fitting the kinetics in this time window with a double exponential decay function, and extrapolating the transient signal beyond 2 ns. The flash photolysis data were then scaled in order to match the extrapolated fraction of unliganded molecules. We should say that, unlike the method proposed by Champion and coworkers ^58^, the current procedure for merging the two different time ranges has some degree of uncertainty since there is about one log _10_ time unit of missing data which may contain kinetic information. Nevertheless, given the shape of the signals, it is expected that this is not a major contribution for the kinetics reported in this work.

**Figure 9.**
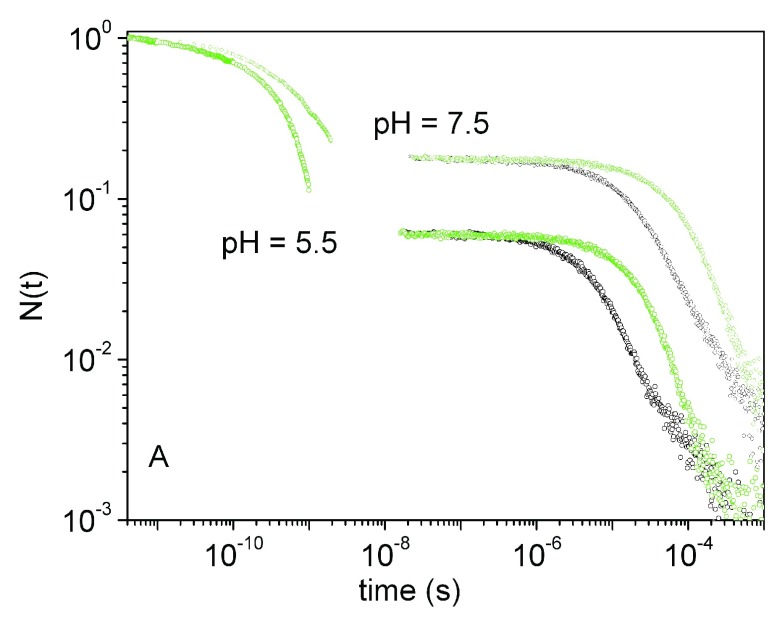
Ligand rebinding kinetics. Complete rebinding kinetics to NP7 at pH 7.5 and pH 5.5 ( *T* = 20°C). The experimental progress curves recorded at 1 CO atm (black open circles) and 0.1 CO atm (green open circles) are reported.

Figure 9 shows the overall rebinding curve for NP7 obtained with this method. From a simple inspection of the progress curves, it is easy to notice that for NP7 the sub-ns kinetics becomes faster and larger when the pH is lowered to 5.5. It is comprised of two kinetic phases, which can be well described by a double exponential decay, whose amplitudes and apparent rates increase at pH 5.5 (not shown). The sub-ns rebinding phase is very similar to the previously described CO rebinding to the related NP4 ^58^. On the longer time scales, the progress curve for NP7 shows the features previously reported, with a heterogeneous bimolecular rebinding which becomes faster as the pH is lowered to 5.5.

**Figure 10.**
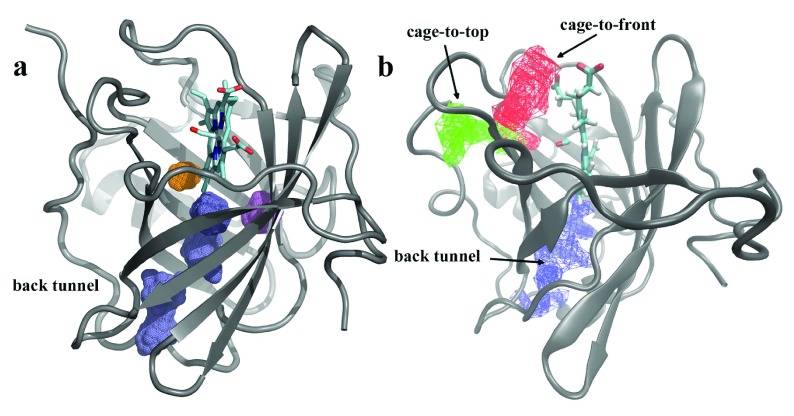
Topological analysis of inner cavities and pathways. ( **a**) Representation of the cavities that form the inner tunnel in NP7 (blue) as well as two additional pockets found around the heme (orange and magenta) identified by the MDpocket analysis. ( **b**) Pathways connecting the heme cavity and the bulk solvent in the closed form of NP7.

Recently we proposed that the reaction progress for NP7 can be described by a microscopic model which takes into account rebinding of photodissociated ligands from internal cavities that are accessible at room temperature ^57^. The current data indicate that in addition to more remote internal cavities, capable of hosting the ligand for a relatively long time, at least one additional temporary docking site exists in the vicinity of the reaction site at the heme, which modulates sub-nanosecond geminate rebinding. From a structural point of view, this assumption is supported by the topological analysis of the inner cavities present in the snapshots sampled along the MD simulation of the closed form of NP7. Thus, Figure 10a shows the three major cavities that shape the tunnel leading from the heme pocket to the back of the protein ^59^ (shown as magenta isocontour) determined by using the MDpocket tool ^60^, and also confirmed by Implicit Ligand Sampling calculations ^61^. Besides the inner tunnel, Figure 10a shows the presence of two additional pockets around the heme. Residues Ile121, Ile123, Leu135 and Ser137 shape the first pocket (orange isocontour), and the side chains of Glu27, Phe43, Phe45 and Leu139 contribute to the second pocket (blue isocontour). Hence, it can be expected that rebinding of photolyzed CO would occur from a variety of transient docking sites, reflecting the topological distribution of pockets located close to the heme (at around ~9 Å from the heme iron) as well as from inner cavities present at the rear of the protein (at around ~22 Å from the heme iron), which can be visited via the inner tunnel present as a distinctive feature in NP7 compared to other NPs.

**Figure 11.**
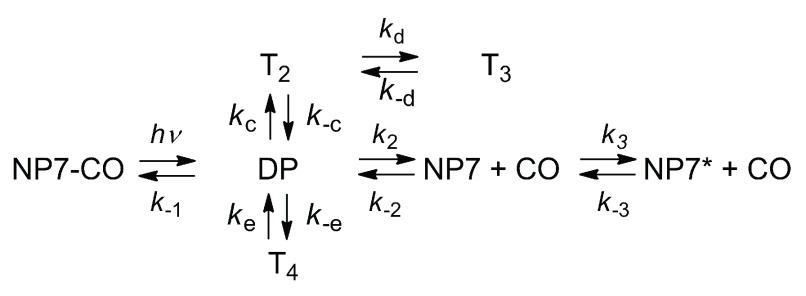
Minimal reaction scheme for the observed rebinding kinetics. Reaction intermediates T _2_, T _3_ and T _4_ denote docking sites inside the protein.

**Figure 12.**
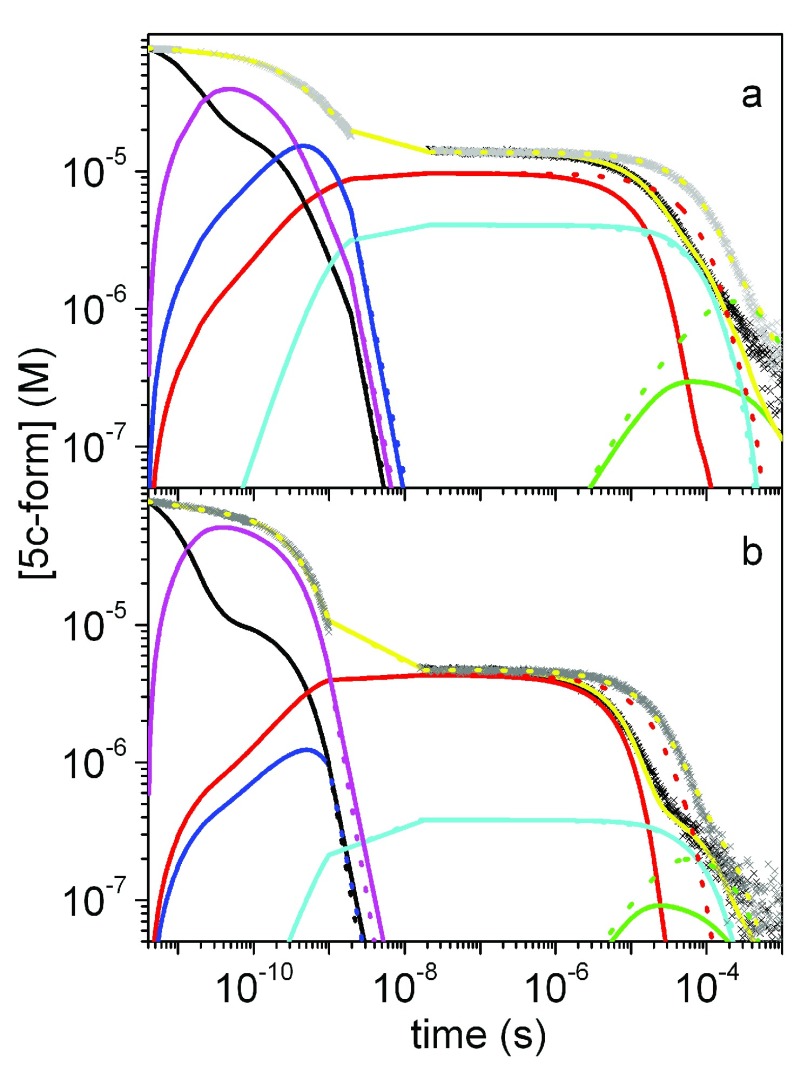
Results of global analysis of the complete course of CO binding kinetics to NP7. ( **A**: pH 7.5, **B**: pH 5.5), at *T* = 20°C and 1 (black) and 0.1 atm (gray). The fits (yellow lines) are superimposed to the experimental data. In the figure, the time course of the other relevant species shown in Figure 8 is reported: DP (black), T _1_ (blue), T _3_ (cyan), T _4_ (magenta), NP (red) and NP7* (green).

On the basis of the previous findings, the reaction scheme previously proposed by us ^59^ is then expanded as shown in Figure 11 to accommodate for transient population T _2_, T _3_ and T _4_ of the ligand in docking sites located nearby the distal pocket (DP), and is found to describe well the rebinding kinetics under the investigated experimental conditions. Sample fits on CO rebinding kinetics to NP7 are reported in Figure 12.

The microscopic rates reported in Table 3 show that exit to the solvent occurs with a remarkably high rate (9 × 10 ^8^ s ^-1^ and 8.2 × 10 ^8^ s ^-1^ at pH 7.5 and 5.5, respectively), which competes, although to a minor extent, with internal rebinding and migration processes. This suggests that the connection between the distal pocket and the solvent must be quite efficient, much more than, for instance, in the case of other proteins with well recognized rather open connections. These include, for instance, myoglobin, for which CO escapes through the His gate at a rate 2.7 × 10 ^6^ s ^-1^ ^62^, and type 1 non symbiotic hemoglobin AHb1 for which exit through the His gate occurs at 5 × 10 ^7^ s ^-1^ ^21, 63^. This nicely parallels the finding of two large and accessible pathways connecting the distal pocket and the solvent (denoted cage-to-top and cage-to-fron in Figure 10b), which can be ascribed to the larger fluctuations of the A-B and G-H loop and widening of the heme cavity in the closed form of wild type NP7 (see Figure 7b).

**Table 3.** Microscopic rate constants from the global fit of the CO rebinding kinetics at 20°C.

	pH 7.5	pH 5.5
*k*_-1_ (10 ^10^ s ^-1^)	0.60±0.02	1.4±0.04
*k*_2_ (10 ^8^ s ^-1^)	9.0±0.4	8.2±0.4
*k*_-2_ (10 ^8^ M ^-1^s ^-1^)	0.6±0.1	1.6±0.2
*k*_3_ (10 ^3^ s ^-1^)	2.0±0.5	2.9±0.7
*k*_-3_ (10 ^3^ s ^-1^)	1.2±0.5	4±1
*k*_c_ (10 ^9^ s ^-1^)	3.7±0.5	0.5±0.1
*k*_-c_ (10 ^9^ s ^-1^)	1.4±0.2	1.2±0.1
*k*_d_ (10 ^8^ s ^-1^)	1.6±0.1	2.1±0.1
*k*_-d_ (10 ^4^ s ^-1^)	1.4±0.4	1.2±0.3
*k*_e_ (10 ^10^ s ^-1^)	4.5±0.7	8.0±1
*k*_-e_ (10 ^10^ s ^-1^)	2.5±0.3	1.9±0.2
*k*_on_ (10 ^8^ M ^-1^s ^-1^)	0.5±0.1	1.5±0.3

Inspection of the microscopic rates in Table 3 also reveals that there are some substantial differences with respect to the values previously determined on the sole basis of nanosecond laser photolysis data ^57^. Inclusion of the ps rebinding phase results in a major increase in rebinding rates, a fact that is not surprising and was anticipated in our previous analysis. The value of the rebinding rate *k*_-1_ is remarkably large compared to, for instance, rebinding to the highly reactive type II truncated Hb from *Thermobifida fusca* (TrHbO), for which rebinding occurs with a rate of 3 × 10 ^8^ s ^-1^ ^64^, a value which is 20 fold smaller than the one for NP7. Mutation of distal cavity residues capable of stabilizing water molecules nearby the binding site lowers the barrier for rebinding and increases the rate to 2 × 10 ^9^ s ^-1^ ^65^, a value which becomes comparable to the value for NP7 at neutral pH, but is still one order of magnitude smaller than observed for NP7 at pH 5.5.

Our results compare well with the average rate constant for CO rebinding to NP4, 4.7 × 10 ^9^ s ^-1^ at pH 7.5 and increasing to 2.1 × 10 ^10^ s ^-1^ at pH 5.5, retrieved by a single distribution model ^58^. In their analysis, Champion and coworkers adopted a statistical model to describe the effect of the strong distortion of the heme in NP4, to explain the observed non-exponential rebinding. In the case of NP7 the ps phase is clearly described by a double exponential decay, and therefore the use of a kinetic model that introduces an additional discrete reaction step appears justified.

Finally, it is important to stress that the *k*_on_ values derived from the current determination of microscopic rate constants reproduces values that well agree with our previous estimates ^57^. The combination of microscopic rates to obtain *k*_on_ is such that the resulting parameter is mostly sensitive to *k*_-2_, whereas it is relatively insensitive to the actual values of *k*_-1_ and *k*_2_ as long as their ratio remains comparable.

Data of membrane attaching nitric oxide transporter nitrophorin 7 Deatiled legends describing each data file can be found in the text file provided.

## Concluding remarks and future directions

The analysis of the X-ray crystal structures of NP7 at low and high pH and with different heme ligands show the existence of a large resemblance in the overall fold, which is also similar to the spatial arrangement found in other NPs. These structures reveal the extensive clustering of Lys side-chains at the protein surface opposite the heme pocket, which is implicated in the charge-stabilized head-to-tail interaction observed in the crystal lattice. Furthermore, it provides a basis to realize the ability of NP7 to bind to negatively charged membranes. However, packing effects also explain the large structural similarity observed for the different ligand-bound and unbound structures, although such a structural similarity does not permit to rationalize the effect of deletion of the N-terminal residues on the binding affinities of certain ligands nor the pH sensitivity of NP7.

The peculiar structural and dynamical properties of NP7 suggest that the mechanism at the basis of the pH sensitivity of this protein exhibits differential features with regard to the one that is operative for the other characterized NP isoforms. The extremely high reactivity of the binding site has the consequence that much of the kinetics is compressed in the sub-nanosecond time scale. In the case of the wt protein, the presence of articulate cavities, endowed with temporary docking sites where photodissociated ligands can migrate, and tunnels connecting the distal pocket with the solvent are proposed to be key determinants for the shape of the observed kinetics. In particular, the additional Leu-Pro-Gly stretch at the N-terminus and the presence of a Glu27 residue in the heme pocket which interferes with the heme vinyl and methyl substituents, both unique among NPs, have profound consequences for the heme pocket structure and dynamics. In particular, the role of Glu27 is still poorly understood and deserves further investigations.

## Data availability

*F1000Research*: Dataset 1. Data of membrane attaching nitric oxide transporter nitrophorin 7, 10.5256/f1000research.6060.d42567 ^66^

## Abbreviations

α _1_m, α _1_-microglobulin; DEA/NO, sodium ( *Z*)-1-( *N*, *N*-diethylamino)diazen-1-ium-1,2-diolate; Hb, hemoglobin; Hm, histamine; ImH, imidazole; IPTG, isopropyl β- d-1-thiogalactopyranosid; L, distal ligand on heme iron; LFP, laser flash photolysis; MALDI, matrix assisted laser desorption ionization; Mb, myoglobin; MD, molecular dynamics; MOPS, 3-( *N*-morpholino)propanesulfonic acid; NOS, nitric oxide synthase; NP, nitrophorin; PDB, Protein Databank of the Research Collaboratory for Structural Bioinformatics (RSCB) at http://www.pdb.org/pdb/home/home.do; RMSD, residual mean square deviation; TOF, time-of-flight; *wt*, wild-type.
