# Supplementary material for: Structure and dynamics of the membrane attaching nitric oxide transporter nitrophorin 7
Source: F1000Res. 2015 Feb 13;4:45. [Version 1] doi: 10.12688/f1000research.6060.1 (PMC4482215; doi:10.12688/f1000research.6060.1)
Supplement: Data of membrane attaching nitric oxide transporter nitrophorin 7 — Deatiled legends describing each data file can be found in the text file provided. [file f1000research-4-6488-s0000.tgz › 63912757-38d2-43f7-9de2-c55ef95641cd_Raw_data.zip!/CV_PDB validation files.zip!/4XMD_val-report-full-annotate_P1.pdf]

Full wwPDB X-ray Structure Validation Report (i)

Jan 15, 2015 – 05:57 AM EST

PDB ID	:	4XMD
Title	:	Crystal structure of nitrophorin 7 from Rhodnius prolixus at pH 7.8
Authors	:	Ogata, H.
Deposited on	:	2015-01-14
Resolution	:	1.60 Å(reported)

DISCLAIMER
This is a preliminary version of the new style of wwPDB validation report.
We welcome your comments at validation@mail.wwpdb.org
A user guide is available at http://wwpdb.org/ValidationPDFNotes.html

CONFIDENTIAL VALIDATION REPORT

The following versions of software and data (see references) were used in the production of this report:

MolProbity	:	4.02b-467
Mogul	:	1.16 November 2013
Xtriage (Phenix)	:	dev-1439
EDS	:	stable24103
Percentile statistics	:	21963
Refmac	:	5.8.0049
CCP4	:	6.3.0 (Settle)
Ideal geometry (proteins)	:	Engh & Huber (2001)
Ideal geometry (DNA, RNA)	:	Parkinson et. al. (1996)
Validation Pipeline (wwPDB-VP)	:	stable24103

1 Overall quality at a glance (i)

The reported resolution of this entry is 1.60 Å.

Percentile scores (ranging between 0-100) for global validation metrics of the entry are shown in the following graphic. The table shows the number of entries on which the scores are based.

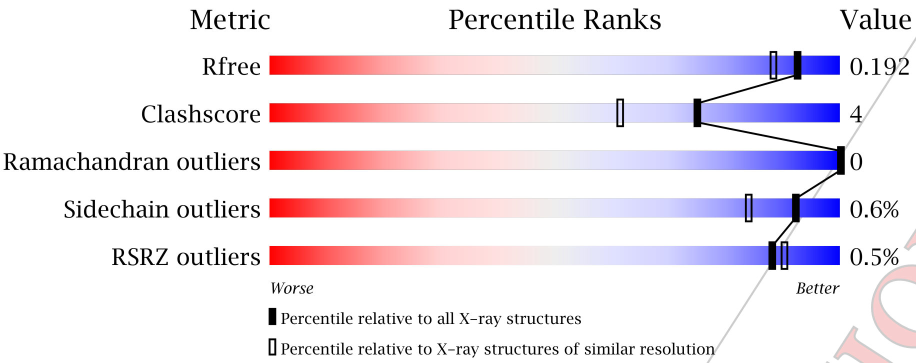

Metric	Whole archive (#Entries)	Similar resolution (#Entries, resolution range(Å))
R_{free}	66092	1872 (1.60-1.60)
Clashscore	79885	2199 (1.60-1.60)
Ramachandran outliers	78287	2126 (1.60-1.60)
Sidechain outliers	78261	2125 (1.60-1.60)
RSRZ outliers	66119	1872 (1.60-1.60)

The table below summarises the geometric issues observed across the polymeric chains and their fit to the electron density. The red, orange, yellow and green segments on the lower bar indicate the fraction of residues that contain outliers for >=3, 2, 1 and 0 types of geometric quality criteria. The upper red bar (where present) indicates the fraction of residues that have poor fit to the electron density.

Mol	Chain	Length	Quality of chain
1	A	184	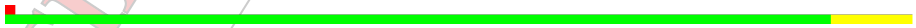

The following table lists non-polymeric compounds that are outliers for geometric or electron-density-fit criteria:

Mol	Type	Chain	Res	Geometry	Electron density
2	HEM	A	201[B]	-	X

CONFIDENTIAL VALIDATION REPORT

2 Entry composition ⓘ

There are 3 unique types of molecules in this entry. The entry contains 1710 atoms, of which 0 are hydrogen and 0 are deuterium.

In the tables below, the ZeroOcc column contains the number of atoms modelled with zero occupancy, the AltConf column contains the number of residues with at least one atom in alternate conformation and the Trace column contains the number of residues modelled with at most 2 atoms.

- Molecule 1 is a protein called Nitrophorin-7.

Mol	Chain	Residues	Atoms					ZeroOcc	AltConf	Trace
			Total	C	N	O	S			
1	A	184	1468	941	241	281	5	0	2	0

- Molecule 2 is PROTOPORPHYRIN IX CONTAINING FE (three-letter code: HEM) (formula: $C_{34}H_{32}FeN_4O_4$).

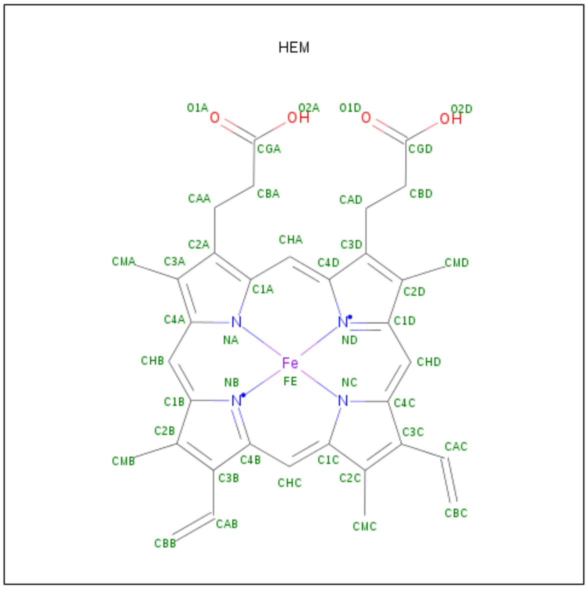

Mol	Chain	Residues	Atoms					ZeroOcc	AltConf
			Total	C	Fe	N	O		
2	A	1	43	34	1	4	4	0	1

- Molecule 3 is water.

Mol	Chain	Residues	Atoms		ZeroOcc	AltConf
			Total	O		
3	A	199	199	199	0	0

CONFIDENTIAL VALIDATION REPORT

3 Residue-property plots (i)

These plots are drawn for all protein, RNA and DNA chains in the entry. The first graphic for a chain summarises the proportions of errors displayed in the second graphic. The second graphic shows the sequence view annotated by issues in geometry and electron density. Residues are color-coded according to the number of geometric quality criteria for which they contain at least one outlier: green = 0, yellow = 1, orange = 2 and red = 3 or more. A red dot above a residue indicates a poor fit to the electron density (RSRZ > 2). Stretches of 2 or more consecutive residues without any outlier are shown as a green connector. Residues present in the sample, but not in the model, are shown in grey.

- Molecule 1: Nitrophorin-7

Chain A: 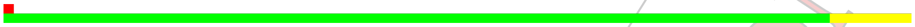

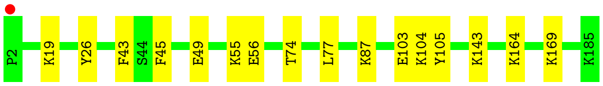

CONFIDENTIAL VALIDATION REPORT

4 Data and refinement statistics (i)

Property	Value	Source
Space group	P 1 21 1	Depositor
Cell constants<br>a, b, c, α, β, γ	38.31Å 66.81Å 39.03Å<br>90.00° 116.87° 90.00°	Depositor
Resolution (Å)	30.42 – 1.60<br>30.43 – 1.60	Depositor<br>EDS
% Data completeness<br>(in resolution range)	99.8 (30.42-1.60)<br>99.8 (30.43-1.60)	Depositor<br>EDS
R_{merge}	0.04	Depositor
R_{sym}	(Not available)	Depositor
$< I/\sigma(I) >$ [1]	2.86 (at 1.60Å)	Xtriage
Refinement program	PHENIX (phenix.refine: dev_1565)	Depositor
R, R_{free}	0.135 , 0.192<br>0.136 , 0.192	Depositor<br>DCC
R_{free} test set	1156 reflections (5.00%)	DCC
Wilson B-factor (Å^2)	17.0	Xtriage
Anisotropy	0.701	Xtriage
Bulk solvent k_{sol}(e/Å^3), B_{sol}(Å^2)	0.36 , 49.3	EDS
Estimated twinning fraction	0.037 for l,-k,h	Xtriage
L-test for twinning	$< \|L\| > = 0.48$, $< L^2 > = 0.31$	Xtriage
Outliers	1 of 23143 reflections (0.004%)	Xtriage
F_o,F_c correlation	0.97	EDS
Total number of atoms	1710	wwPDB-VP
Average B, all atoms (Å^2)	28.0	wwPDB-VP

Xtriage's analysis on translational NCS is as follows: *The largest off-origin peak in the Patterson function is 8.52% of the height of the origin peak. No significant pseudotranslation is detected.*

[1]Intensities estimated from amplitudes.

CONFIDENTIAL VALIDATION REPORT

5 Model quality (i)

5.1 Standard geometry (i)

Bond lengths and bond angles in the following residue types are not validated in this section: HEM

The Z score for a bond length (or angle) is the number of standard deviations the observed value is removed from the expected value. A bond length (or angle) with $|Z| > 5$ is considered an outlier worth inspection. RMSZ is the root-mean-square of all Z scores of the bond lengths (or angles).

Mol	Chain	Bond lengths		Bond angles	
		RMSZ	$\#|Z| > 5$	RMSZ	$\#|Z| > 5$
1	A	0.33	0/1500	0.50	0/2021

There are no bond length outliers.

There are no bond angle outliers.

There are no chirality outliers.

There are no planarity outliers.

5.2 Close contacts (i)

In the following table, the Non-H and H(model) columns list the number of non-hydrogen atoms and hydrogen atoms in the chain respectively. The H(added) column lists the number of hydrogens added by MolProbity. The Clashes column lists the number of clashes within the asymmetric unit, and the number in parentheses is this value normalized per 1000 atoms of the molecule in the chain. The Symm-Clashes column gives symmetry related clashes, in the same way as for the Clashes column.

Mol	Chain	Non-H	H(model)	H(added)	Clashes	Symm-Clashes
1	A	1468	0	1465	12	0
2	A	43	0	30	2	0
3	A	199	0	0	2	0
All	All	1710	0	1495	12	0

Clashscore is defined as the number of clashes calculated for the entry per 1000 atoms (including hydrogens) of the entry. The overall clashscore for this entry is 4.

All (12) close contacts within the same asymmetric unit are listed below.

Atom-1	Atom-2	Distance(Å)	Clash(Å)
1:A:55:LYS:HG2	1:A:74[B]:THR:HG22	1.84	0.59

Continued on next page...

CONFIDENTIAL VALIDATION REPORT

Continued from previous page...

Atom-1	Atom-2	Distance(Å)	Clash(Å)
1:A:169:LYS:NZ	3:A:301:HOH:O	2.34	0.58
1:A:43:PHE:CE2	2:A:201[B]:HEM:HMC3	2.41	0.55
1:A:87:LYS:HE2	1:A:103:GLU:O	2.07	0.55
1:A:74[B]:THR:HG21	3:A:362:HOH:O	2.10	0.52
1:A:26:TYR:CE1	1:A:143:LYS:HE2	2.49	0.48
1:A:104:LYS:HE2	1:A:105:TYR:CZ	2.49	0.48
1:A:19:LYS:HD3	1:A:19:LYS:HA	1.74	0.46
1:A:104:LYS:HE2	1:A:105:TYR:CE2	2.52	0.44
1:A:49:GLU:HG3	1:A:77:LEU:HD22	2.00	0.43
1:A:56:GLU:OE2	2:A:201[B]:HEM:HBC1	2.19	0.42
1:A:164:LYS:HB3	1:A:164:LYS:HE2	1.76	0.42

There are no symmetry-related clashes.

5.3 Torsion angles

5.3.1 Protein backbone (i)

In the following table, the Percentiles column shows the percent Ramachandran outliers of the chain as a percentile score with respect to all X-ray entries followed by that with respect to entries of similar resolution.

The Analysed column shows the number of residues for which the backbone conformation was analysed, and the total number of residues.

Mol	Chain	Analysed	Favoured	Allowed	Outliers	Percentiles
1	A	183/184 (100%)	180 (98%)	3 (2%)	0	100 100

There are no Ramachandran outliers to report.

5.3.2 Protein sidechains (i)

In the following table, the Percentiles column shows the percent sidechain outliers of the chain as a percentile score with respect to all X-ray entries followed by that with respect to entries of similar resolution. The Analysed column shows the number of residues for which the sidechain conformation was analysed, and the total number of residues.

Mol	Chain	Analysed	Rotameric	Outliers	Percentiles
1	A	163/162 (101%)	162 (99%)	1 (1%)	92 84

All (1) residues with a non-rotameric sidechain are listed below:

CONFIDENTIAL VALIDATION REPORT

Mol	Chain	Res	Type
1	A	45	PHE

Some sidechains can be flipped to improve hydrogen bonding and reduce clashes. There are no such sidechains identified.

5.3.3 RNA ⓘ

There are no RNA chains in this entry.

5.4 Non-standard residues in protein, DNA, RNA chains ⓘ

There are no non-standard protein/DNA/RNA residues in this entry.

5.5 Carbohydrates ⓘ

There are no carbohydrates in this entry.

5.6 Ligand geometry ⓘ

1 ligand is modelled in this entry.

In the following table, the Counts columns list the number of bonds (or angles) for which Mogul statistics could be retrieved, the number of bonds (or angles) that are observed in the model and the number of bonds (or angles) that are defined in the chemical component dictionary. The Link column lists molecule types, if any, to which the group is linked. The Z score for a bond length (or angle) is the number of standard deviations the observed value is removed from the expected value. A bond length (or angle) with $|Z| > 2$ is considered an outlier worth inspection. RMSZ is the root-mean-square of all Z scores of the bond lengths (or angles).

Mol	Type	Chain	Res	Link	Bond lengths			Bond angles		
					Counts	RMSZ	$\#\|Z\| > 2$	Counts	RMSZ	$\#\|Z\| > 2$
2	HEM	A	201[B]	1,3	42,50,50	3.52	13 (30%)	27,82,82	1.35	4 (14%)

In the following table, the Chirals column lists the number of chiral outliers, the number of chiral centers analysed, the number of these observed in the model and the number defined in the chemical component dictionary. Similar counts are reported in the Torsion and Rings columns. '-' means no outliers of that kind were identified.

Mol	Type	Chain	Res	Link	Chirals	Torsions	Rings
2	HEM	A	201[B]	1,3	-	0/14/114/114	0/0/8/8

CONFIDENTIAL VALIDATION REPORT

All (13) bond length outliers are listed below:

Mol	Chain	Res	Type	Atoms	Z	Observed(Å)	Ideal(Å)
2	A	201[B]	HEM	C3C-C2C	-13.54	1.34	1.45
2	A	201[B]	HEM	C3B-C2B	-10.82	1.35	1.45
2	A	201[B]	HEM	CMB-C2B	5.68	1.54	1.45
2	A	201[B]	HEM	CMC-C2C	5.61	1.54	1.45
2	A	201[B]	HEM	CMD-C2D	5.30	1.54	1.45
2	A	201[B]	HEM	C3C-CAC	4.61	1.55	1.40
2	A	201[B]	HEM	C3B-CAB	4.53	1.55	1.40
2	A	201[B]	HEM	C3D-C2D	3.81	1.53	1.43
2	A	201[B]	HEM	FE-ND	3.13	2.08	1.95
2	A	201[B]	HEM	FE-NB	2.83	2.05	1.95
2	A	201[B]	HEM	FE-NC	2.81	2.07	1.95
2	A	201[B]	HEM	C4D-ND	2.49	1.40	1.33
2	A	201[B]	HEM	C1D-ND	2.46	1.39	1.33

All (4) bond angle outliers are listed below:

Mol	Chain	Res	Type	Atoms	Z	Observed(^{o})	Ideal(^{o})
2	A	201[B]	HEM	CBA-CAA-C2A	-3.30	107.13	112.63
2	A	201[B]	HEM	CAD-CBD-CGD	-2.54	108.73	113.53
2	A	201[B]	HEM	C3A-C4A-NA	-2.05	108.13	109.50
2	A	201[B]	HEM	CMA-C3A-C4A	-2.00	125.39	128.46

There are no chirality outliers.

There are no torsion outliers.

There are no ring outliers.

5.7 Other polymers (i)

There are no such residues in this entry.

5.8 Polymer linkage issues

There are no chain breaks in this entry.

CONFIDENTIAL VALIDATION REPORT

6 Fit of model and data (i)

6.1 Protein, DNA and RNA chains (i)

In the following table, the column labelled '#RSRZ> 2' contains the number (and percentage) of RSRZ outliers, followed by percent RSRZ outliers for the chain as percentile scores relative to all X-ray entries and entries of similar resolution. The OWAB column contains the minimum, median, 95th percentile and maximum values of the occupancy-weighted average B-factor per residue. The column labelled 'Q< 0.9' lists the number of (and percentage) of residues with an average occupancy less than 0.9.

Mol	Chain	Analysed	<RSRZ>	#RSRZ>2			OWAB(Å^2)	Q<0.9
1	A	184/184 (100%)	-0.41	1 (0%)	88	90	14, 25, 46, 62	1 (0%)

All (1) RSRZ outliers are listed below:

Mol	Chain	Res	Type	RSRZ
1	A	2	PRO	3.3

6.2 Non-standard residues in protein, DNA, RNA chains (i)

There are no non-standard protein/DNA/RNA residues in this entry.

6.3 Carbohydrates (i)

There are no carbohydrates in this entry.

6.4 Ligands (i)

In the following table, the Atoms column lists the number of modelled atoms in the group and the number defined in the chemical component dictionary. LLDF column lists the quality of electron density of the group with respect to its neighbouring residues in protein, DNA or RNA chains. The B-factors column lists the minimum, median, 95th percentile and maximum values of B factors of atoms in the group. The column labelled 'Q< 0.9' lists the number of atoms with occupancy less than 0.9.

Mol	Type	Chain	Res	Atoms	RSR	LLDF	B-factors(Å^2)	Q<0.9
2	HEM	A	201[B]	43/43	0.18	3.49	23,32,43,46	43

CONFIDENTIAL VALIDATION REPORT

6.5 Other polymers (i)

There are no such residues in this entry.

CONFIDENTIAL VALIDATION REPORT
