# Supplementary material for: Structure and dynamics of the membrane attaching nitric oxide transporter nitrophorin 7
Source: F1000Res. 2015 Feb 13;4:45. [Version 1] doi: 10.12688/f1000research.6060.1 (PMC4482215; doi:10.12688/f1000research.6060.1)
Supplement: Data of membrane attaching nitric oxide transporter nitrophorin 7 — Deatiled legends describing each data file can be found in the text file provided. [file f1000research-4-6488-s0000.tgz › 63912757-38d2-43f7-9de2-c55ef95641cd_Raw_data.zip!/CV_PDB validation files.zip!/4XMF_val-report-full-annotate_P1.pdf]

# Full wwPDB X-ray Structure Validation Report (i)

Jan 15, 2015 – 06:32 AM EST

| | | |
|---|---|---|
| PDB ID | : | 4XMF |
| Title | : | Crystal structure of nitrophorin 7 from Rhodnius prolixus at pH 7.8 complexed with histamine |
| Authors | : | Ogata, H. |
| Deposited on | : | 2015-01-14 |
| Resolution | : | 1.60 Å(reported) |

**DISCLAIMER**
This is a preliminary version of the new style of wwPDB validation report.
We welcome your comments at validation@mail.wwpdb.org
A user guide is available at http://wwpdb.org/ValidationPDFNotes.html

CONFIDENTIAL VALIDATION REPORT

The following versions of software and data (see references) were used in the production of this report:

| | | |
|---|---|---|
| MolProbity | : | 4.02b-467 |
| Mogul | : | 1.16 November 2013 |
| Xtriage (Phenix) | : | dev-1439 |
| EDS | : | stable24103 |
| Percentile statistics | : | 21963 |
| Refmac | : | 5.8.0049 |
| CCP4 | : | 6.3.0 (Settle) |
| Ideal geometry (proteins) | : | Engh & Huber (2001) |
| Ideal geometry (DNA, RNA) | : | Parkinson et. al. (1996) |
| Validation Pipeline (wwPDB-VP) | : | stable24103 |

# 1 Overall quality at a glance (i)

The reported resolution of this entry is 1.60 Å.

Percentile scores (ranging between 0-100) for global validation metrics of the entry are shown in the following graphic. The table shows the number of entries on which the scores are based.

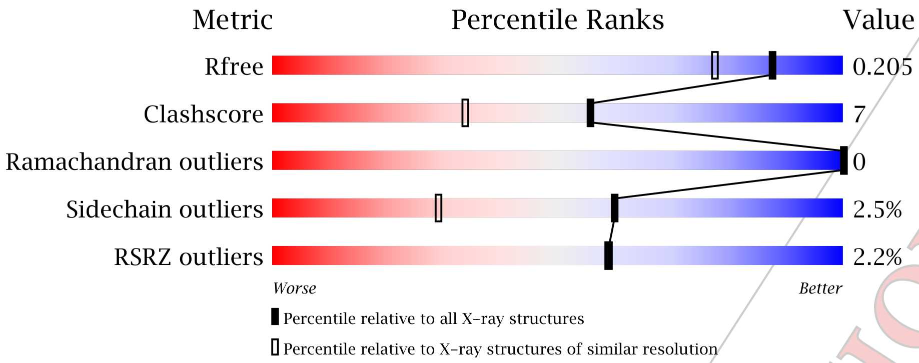


| Metric | Whole archive (#Entries) | Similar resolution (#Entries, resolution range(Å)) |
|---|---|---|
| $R_{free}$ | 66092 | 1872 (1.60-1.60) |
| Clashscore | 79885 | 2199 (1.60-1.60) |
| Ramachandran outliers | 78287 | 2126 (1.60-1.60) |
| Sidechain outliers | 78261 | 2125 (1.60-1.60) |
| RSRZ outliers | 66119 | 1872 (1.60-1.60) |

The table below summarises the geometric issues observed across the polymeric chains and their fit to the electron density. The red, orange, yellow and green segments on the lower bar indicate the fraction of residues that contain outliers for >=3, 2, 1 and 0 types of geometric quality criteria. The upper red bar (where present) indicates the fraction of residues that have poor fit to the electron density.

| Mol | Chain | Length | Quality of chain |
|---|---|---|---|
| 1 | A | 184 | |

The following table lists non-polymeric compounds that are outliers for geometric or electron-density-fit criteria:

| Mol | Type | Chain | Res | Geometry | Electron density |
|---|---|---|---|---|---|
| 3 | HSM | A | 202 | - | X |

CONFIDENTIAL VALIDATION REPORT

# 2 Entry composition (i)

There are 4 unique types of molecules in this entry. The entry contains 1638 atoms, of which 0 are hydrogen and 0 are deuterium.

In the tables below, the ZeroOcc column contains the number of atoms modelled with zero occupancy, the AltConf column contains the number of residues with at least one atom in alternate conformation and the Trace column contains the number of residues modelled with at most 2 atoms.

- Molecule 1 is a protein called Nitrophorin-7.

| Mol | Chain | Residues | Atoms | | | | | ZeroOcc | AltConf | Trace |
|---|---|---|---|---|---|---|---|---|---|---|
| | | | Total | C | N | O | S | | | |
| 1 | A | 184 | 1461 | 937 | 240 | 279 | 5 | 0 | 0 | 0 |

- Molecule 2 is PROTOPORPHYRIN IX CONTAINING FE (three-letter code: HEM) (formula: $C_{34}H_{32}FeN_4O_4$).

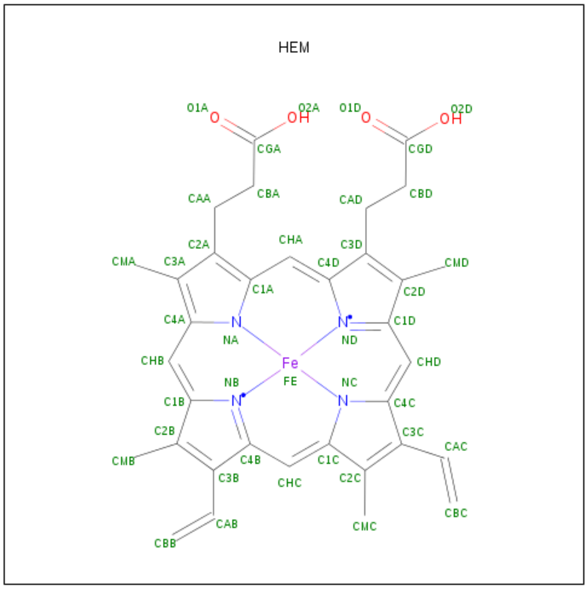


| Mol | Chain | Residues | Atoms | | | | | ZeroOcc | AltConf |
|---|---|---|---|---|---|---|---|---|---|
| | | | Total | C | Fe | N | O | | |
| 2 | A | 1 | 43 | 34 | 1 | 4 | 4 | 0 | 0 |

- Molecule 3 is HISTAMINE (three-letter code: HSM) (formula: $C_5H_9N_3$).

CONFIDENTIAL - VALIDATION REPORT

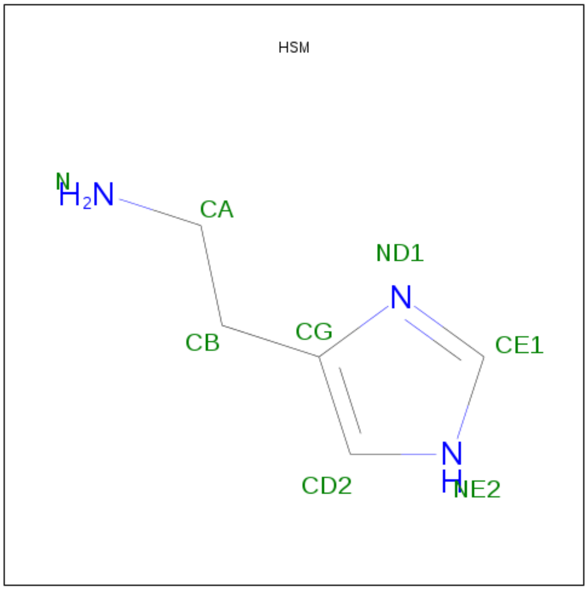


| Mol | Chain | Residues | Atoms | | | ZeroOcc | AltConf |
|---|---|---|---|---|---|---|---|
| | | | Total | C | N | | |
| 3 | A | 1 | 8 | 5 | 3 | 0 | 0 |

- Molecule 4 is water.

| Mol | Chain | Residues | Atoms | | ZeroOcc | AltConf |
|---|---|---|---|---|---|---|
| | | | Total | O | | |
| 4 | A | 126 | 126 | 126 | 0 | 0 |

CONFIDENTIAL VALIDATION REPORT

# 3 Residue-property plots (i)

These plots are drawn for all protein, RNA and DNA chains in the entry. The first graphic for a chain summarises the proportions of errors displayed in the second graphic. The second graphic shows the sequence view annotated by issues in geometry and electron density. Residues are color-coded according to the number of geometric quality criteria for which they contain at least one outlier: green = 0, yellow = 1, orange = 2 and red = 3 or more. A red dot above a residue indicates a poor fit to the electron density (RSRZ > 2). Stretches of 2 or more consecutive residues without any outlier are shown as a green connector. Residues present in the sample, but not in the model, are shown in grey.

- Molecule 1: Nitrophorin-7

Chain A: 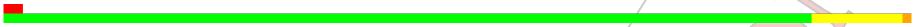

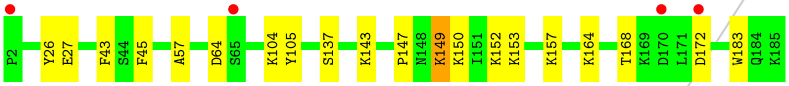


CONFIDENTIAL VALIDATION REPORT

# 4 Data and refinement statistics

| Property | Value | Source |
|---|---|---|
| Space group | P 1 21 1 | Depositor |
| Cell constants<br>a, b, c, $\alpha$, $\beta$, $\gamma$ | 38.34Å 66.72Å 38.83Å<br>90.00° 116.87° 90.00° | Depositor |
| Resolution (Å) | 30.74 – 1.60<br>30.74 – 1.60 | Depositor<br>EDS |
| % Data completeness<br>(in resolution range) | 97.3 (30.74-1.60)<br>97.3 (30.74-1.60) | Depositor<br>EDS |
| $R_{merge}$ | 0.07 | Depositor |
| $R_{sym}$ | (Not available) | Depositor |
| $< I/\sigma(I) >$ [1] | 2.25 (at 1.60Å) | Xtriage |
| Refinement program | PHENIX (phenix.refine: 2011_08_24_1020) | Depositor |
| R, $R_{free}$ | 0.158 , 0.207<br>0.154 , 0.205 | Depositor<br>DCC |
| $R_{free}$ test set | 1120 reflections (5.00%) | DCC |
| Wilson B-factor (Å$^2$) | 14.7 | Xtriage |
| Anisotropy | 0.654 | Xtriage |
| Bulk solvent $k_{sol}$(e/Å$^3$), $B_{sol}$(Å$^2$) | 0.35 , 36.9 | EDS |
| Estimated twinning fraction | 0.037 for l,-k,h | Xtriage |
| L-test for twinning | $< \|L\| >$ = 0.50, $< L^2 >$ = 0.33 | Xtriage |
| Outliers | 0 of 22417 reflections | Xtriage |
| $F_o$,$F_c$ correlation | 0.97 | EDS |
| Total number of atoms | 1638 | wwPDB-VP |
| Average B, all atoms (Å$^2$) | 22.0 | wwPDB-VP |

Xtriage's analysis on translational NCS is as follows: *The largest off-origin peak in the Patterson function is 8.38% of the height of the origin peak. No significant pseudotranslation is detected.*

[1] Intensities estimated from amplitudes.

CONFIDENTIAL VALIDATION REPORT

# 5 Model quality (i)

## 5.1 Standard geometry (i)

Bond lengths and bond angles in the following residue types are not validated in this section: HEM, HSM

The Z score for a bond length (or angle) is the number of standard deviations the observed value is removed from the expected value. A bond length (or angle) with $|Z| > 5$ is considered an outlier worth inspection. RMSZ is the root-mean-square of all Z scores of the bond lengths (or angles).

| Mol | Chain | Bond lengths | | Bond angles | |
|---|---|---|---|---|---|
| | | RMSZ | $\#\|Z\| > 5$ | RMSZ | $\#\|Z\| > 5$ |
| 1 | A | 0.34 | 0/1493 | 0.51 | 0/2011 |

There are no bond length outliers.

There are no bond angle outliers.

There are no chirality outliers.

There are no planarity outliers.

## 5.2 Close contacts (i)

In the following table, the Non-H and H(model) columns list the number of non-hydrogen atoms and hydrogen atoms in the chain respectively. The H(added) column lists the number of hydrogens added by MolProbity. The Clashes column lists the number of clashes within the asymmetric unit, and the number in parentheses is this value normalized per 1000 atoms of the molecule in the chain. The Symm-Clashes column gives symmetry related clashes, in the same way as for the Clashes column.

| Mol | Chain | Non-H | H(model) | H(added) | Clashes | Symm-Clashes |
|---|---|---|---|---|---|---|
| 1 | A | 1461 | 0 | 1460 | 15 | 0 |
| 2 | A | 43 | 0 | 30 | 9 | 0 |
| 3 | A | 8 | 0 | 8 | 4 | 0 |
| 4 | A | 126 | 0 | 0 | 2 | 0 |
| All | All | 1638 | 0 | 1498 | 20 | 0 |

Clashscore is defined as the number of clashes calculated for the entry per 1000 atoms (including hydrogens) of the entry. The overall clashscore for this entry is 7.

All (20) close contacts within the same asymmetric unit are listed below.

CONFIDENTIAL VALIDATION REPORT

| Atom-1 | Atom-2 | Distance(Å) | Clash(Å) |
|---|---|---|---|
| 3:A:202:HSM:N | 4:A:301:HOH:O | 2.15 | 0.79 |
| 1:A:27:GLU:OE2 | 1:A:168:THR:OG1 | 2.03 | 0.77 |
| 2:A:201:HEM:HHC | 2:A:201:HEM:HBB2 | 1.68 | 0.75 |
| 2:A:201:HEM:C4A | 3:A:202:HSM:HD2 | 2.32 | 0.65 |
| 1:A:150:LYS:NZ | 4:A:302:HOH:O | 2.35 | 0.59 |
| 1:A:27:GLU:HG3 | 2:A:201:HEM:HBB1 | 1.85 | 0.59 |
| 1:A:137:SER:OG | 2:A:201:HEM:HBB1 | 2.12 | 0.49 |
| 1:A:149:LYS:NZ | 1:A:149:LYS:H | 2.14 | 0.46 |
| 1:A:153:LYS:O | 1:A:157:LYS:HG3 | 2.16 | 0.46 |
| 1:A:43:PHE:CZ | 2:A:201:HEM:HMC3 | 2.51 | 0.45 |
| 1:A:147:PRO:HG2 | 1:A:152:LYS:HE3 | 1.99 | 0.44 |
| 2:A:201:HEM:C4A | 3:A:202:HSM:CD2 | 3.00 | 0.44 |
| 1:A:27:GLU:HG3 | 2:A:201:HEM:CBB | 2.47 | 0.44 |
| 1:A:104:LYS:HE2 | 1:A:105:TYR:CZ | 2.52 | 0.44 |
| 1:A:26:TYR:CZ | 1:A:143:LYS:HE2 | 2.54 | 0.43 |
| 2:A:201:HEM:NA | 3:A:202:HSM:CD2 | 2.81 | 0.42 |
| 1:A:43:PHE:CE2 | 2:A:201:HEM:HMC3 | 2.53 | 0.42 |
| 1:A:164:LYS:HB3 | 1:A:164:LYS:HE2 | 1.88 | 0.42 |
| 1:A:26:TYR:CE1 | 1:A:143:LYS:HE2 | 2.55 | 0.41 |
| 1:A:57:ALA:HB2 | 1:A:183:TRP:CE3 | 2.56 | 0.41 |

There are no symmetry-related clashes.

## 5.3 Torsion angles

### 5.3.1 Protein backbone (i)

In the following table, the Percentiles column shows the percent Ramachandran outliers of the chain as a percentile score with respect to all X-ray entries followed by that with respect to entries of similar resolution.

The Analysed column shows the number of residues for which the backbone conformation was analysed, and the total number of residues.

| Mol | Chain | Analysed | Favoured | Allowed | Outliers | Percentiles | |
|---|---|---|---|---|---|---|---|
| 1 | A | 182/184 (99%) | 180 (99%) | 2 (1%) | 0 | 100 | 100 |

There are no Ramachandran outliers to report.

CONFIDENTIAL VALIDATION REPORT

### 5.3.2 Protein sidechains (i)

In the following table, the Percentiles column shows the percent sidechain outliers of the chain as a percentile score with respect to all X-ray entries followed by that with respect to entries of similar resolution. The Analysed column shows the number of residues for which the sidechain conformation was analysed, and the total number of residues.

| Mol | Chain | Analysed | Rotameric | Outliers | Percentiles | |
|---|---|---|---|---|---|---|
| 1 | A | 162/162 (100%) | 158 (98%) | 4 (2%) | 60 | 29 |

All (4) residues with a non-rotameric sidechain are listed below:

| Mol | Chain | Res | Type |
|---|---|---|---|
| 1 | A | 45 | PHE |
| 1 | A | 64 | ASP |
| 1 | A | 149 | LYS |
| 1 | A | 172 | ASP |

Some sidechains can be flipped to improve hydrogen bonding and reduce clashes. There are no such sidechains identified.

### 5.3.3 RNA (i)

There are no RNA chains in this entry.

## 5.4 Non-standard residues in protein, DNA, RNA chains (i)

There are no non-standard protein/DNA/RNA residues in this entry.

## 5.5 Carbohydrates (i)

There are no carbohydrates in this entry.

## 5.6 Ligand geometry (i)

2 ligands are modelled in this entry.

In the following table, the Counts columns list the number of bonds (or angles) for which Mogul statistics could be retrieved, the number of bonds (or angles) that are observed in the model and the number of bonds (or angles) that are defined in the chemical component dictionary. The Link column lists molecule types, if any, to which the group is linked. The Z score for a bond length (or angle) is the number of standard deviations the observed value is removed from the expected

CONFIDENTIAL VALIDATION REPORT

value. A bond length (or angle) with $|Z| > 2$ is considered an outlier worth inspection. RMSZ is the root-mean-square of all Z scores of the bond lengths (or angles).

| Mol | Type | Chain | Res | Link | Bond lengths | | | Bond angles | | |
|---|---|---|---|---|---|---|---|---|---|---|
| | | | | | Counts | RMSZ | $\#\|Z\| > 2$ | Counts | RMSZ | $\#\|Z\| > 2$ |
| 2 | HEM | A | 201 | 1,3 | 42,50,50 | 3.64 | 12 (28%) | 27,82,82 | 1.36 | 3 (11%) |
| 3 | HSM | A | 202 | 2 | 8,8,8 | 2.03 | 2 (25%) | 9,9,9 | 2.35 | 3 (33%) |

In the following table, the Chirals column lists the number of chiral outliers, the number of chiral centers analysed, the number of these observed in the model and the number defined in the chemical component dictionary. Similar counts are reported in the Torsion and Rings columns. '-' means no outliers of that kind were identified.

| Mol | Type | Chain | Res | Link | Chirals | Torsions | Rings |
|---|---|---|---|---|---|---|---|
| 2 | HEM | A | 201 | 1,3 | - | 0/14/114/114 | 0/0/8/8 |
| 3 | HSM | A | 202 | 2 | - | 2/3/3/3 | 0/1/1/1 |

All (14) bond length outliers are listed below:

| Mol | Chain | Res | Type | Atoms | Z | Observed(Å) | Ideal(Å) |
|---|---|---|---|---|---|---|---|
| 2 | A | 201 | HEM | C3C-C2C | -13.54 | 1.34 | 1.45 |
| 2 | A | 201 | HEM | C3B-C2B | -12.67 | 1.33 | 1.45 |
| 2 | A | 201 | HEM | CMC-C2C | 5.65 | 1.54 | 1.45 |
| 2 | A | 201 | HEM | CMB-C2B | 5.53 | 1.54 | 1.45 |
| 2 | A | 201 | HEM | CMD-C2D | 5.44 | 1.54 | 1.45 |
| 2 | A | 201 | HEM | C3C-CAC | 4.55 | 1.55 | 1.40 |
| 2 | A | 201 | HEM | C3B-CAB | 4.47 | 1.55 | 1.40 |
| 3 | A | 202 | HSM | CD2-CG | 3.95 | 1.41 | 1.36 |
| 2 | A | 201 | HEM | C3D-C2D | 3.78 | 1.53 | 1.43 |
| 2 | A | 201 | HEM | FE-ND | 3.47 | 2.09 | 1.95 |
| 3 | A | 202 | HSM | CD2-NE2 | -3.37 | 1.32 | 1.36 |
| 2 | A | 201 | HEM | C1D-ND | 2.60 | 1.40 | 1.33 |
| 2 | A | 201 | HEM | C4D-ND | 2.48 | 1.40 | 1.33 |
| 2 | A | 201 | HEM | FE-NB | 2.44 | 2.04 | 1.95 |

All (6) bond angle outliers are listed below:

| Mol | Chain | Res | Type | Atoms | Z | Observed($^{o}$) | Ideal($^{o}$) |
|---|---|---|---|---|---|---|---|
| 3 | A | 202 | HSM | CB-CG-ND1 | 5.21 | 127.56 | 121.87 |
| 2 | A | 201 | HEM | CBA-CAA-C2A | -3.89 | 106.16 | 112.63 |
| 3 | A | 202 | HSM | CB-CG-CD2 | -3.60 | 125.75 | 131.17 |
| 2 | A | 201 | HEM | CBD-CAD-C3D | -2.37 | 109.37 | 114.51 |
| 2 | A | 201 | HEM | CAD-CBD-CGD | -2.06 | 109.64 | 113.53 |
| 3 | A | 202 | HSM | CB-CA-N | 2.03 | 117.00 | 112.15 |

CONFIDENTIAL VALIDATION REPORT

There are no chirality outliers.

All (2) torsion outliers are listed below:

| Mol | Chain | Res | Type | Atoms |
|---|---|---|---|---|
| 3 | A | 202 | HSM | CD2-CG-CB-CA |
| 3 | A | 202 | HSM | CA-CB-CG-ND1 |

There are no ring outliers.

## 5.7 Other polymers (i)

There are no such residues in this entry.

## 5.8 Polymer linkage issues

There are no chain breaks in this entry.

CONFIDENTIAL VALIDATION REPORT

# 6 Fit of model and data

## 6.1 Protein, DNA and RNA chains

In the following table, the column labelled '#RSRZ> 2' contains the number (and percentage) of RSRZ outliers, followed by percent RSRZ outliers for the chain as percentile scores relative to all X-ray entries and entries of similar resolution. The OWAB column contains the minimum, median, $95^{th}$ percentile and maximum values of the occupancy-weighted average B-factor per residue. The column labelled 'Q< 0.9' lists the number of (and percentage) of residues with an average occupancy less than 0.9.

| Mol | Chain | Analysed | <RSRZ> | #RSRZ>2 | | | OWAB(Å$^2$) | Q<0.9 |
|---|---|---|---|---|---|---|---|---|
| 1 | A | 184/184 (100%) | -0.32 | 4 (2%) | 59 | 59 | 9, 19, 39, 54 | 1 (0%) |

All (4) RSRZ outliers are listed below:

| Mol | Chain | Res | Type | RSRZ |
|---|---|---|---|---|
| 1 | A | 2 | PRO | 2.6 |
| 1 | A | 170 | ASP | 2.5 |
| 1 | A | 172 | ASP | 2.2 |
| 1 | A | 65 | SER | 2.2 |

## 6.2 Non-standard residues in protein, DNA, RNA chains

There are no non-standard protein/DNA/RNA residues in this entry.

## 6.3 Carbohydrates

There are no carbohydrates in this entry.

## 6.4 Ligands

In the following table, the Atoms column lists the number of modelled atoms in the group and the number defined in the chemical component dictionary. LLDF column lists the quality of electron density of the group with respect to its neighbouring residues in protein, DNA or RNA chains. The B-factors column lists the minimum, median, $95^{th}$ percentile and maximum values of B factors of atoms in the group. The column labelled 'Q< 0.9' lists the number of atoms with occupancy less than 0.9.

| Mol | Type | Chain | Res | Atoms | RSR | LLDF | B-factors(Å$^2$) | Q<0.9 |
|---|---|---|---|---|---|---|---|---|
| 3 | HSM | A | 202 | 8/8 | 0.24 | 9.16 | 27,29,31,34 | 8 |
| 2 | HEM | A | 201 | 43/43 | 0.12 | 1.27 | 18,24,40,43 | 43 |

CONFIDENTIAL VALIDATION REPORT

### 6.5 Other polymers (i)

There are no such residues in this entry.

CONFIDENTIAL VALIDATION REPORT
