# Supplementary material for: Structure and dynamics of the membrane attaching nitric oxide transporter nitrophorin 7
Source: F1000Res. 2015 Feb 13;4:45. [Version 1] doi: 10.12688/f1000research.6060.1 (PMC4482215; doi:10.12688/f1000research.6060.1)
Supplement: Peer review history

# Peer review: Referee response for version 1

**DOI:** 10.5256/f1000research.6488.r9066

**Published:** 2015-07-27

**Authors:** Antonio Cupane, Matteo Levantino

**Affiliations:** 1 Department of Physics and Chemistry, University of Palermo, Palermo, Italy

The article by Knipp *et al*. reports the results of an extensive investigation of the nitrophorin isoform NP7. The authors have resolved the X-ray crystallographic structure of NP7 at different pH values and ligation states, measured the ligand rebinding kinetics after laser photolysis of CO bound NP7 at different pH values and performed MD simulations on different NP7 conformations to assess the degree of flexibility of different protein domains and the topology of inner protein cavities that are shown to play a significant role in the binding of ligands to the protein.

The presented results are new and significant to our understanding of the structure-function-dynamics relationship in the NP7 nitrophorin. However, the paper would benefit from a more clear discussion of an important aspect that emerges from the results presented by authors: the charge distribution across NP7, which peculiarly determines the ability of this particular nitrophorin isoform to bind negatively charged membranes, is likely to significantly affect both the way NP7 molecules are arranged in the crystalline state and their overall 3D structure in the crystal. As stated by the authors, the surprisingly small differences between the crystal structure of NP7-H2O at pH 5.8 and 7.8 could be due to the overwhelming contribution of electrostatic interactions between neighboring proteins within the crystal. The authors refrained from using their NP7 X-ray structures as starting points for their MD simulations since they admittedly do not account for the conformational change expected to occur upon increase of the pH from 5.8 to 7.8. Overall, these findings seem to indicate that, in the case of NP7 nitrophorin, the structural models retrieved from X-ray crystallography fail to accurately describe the 3D structure of the protein in solution.

It would be worth to better clarify and discuss this point in the paper.

A further important result of the paper comes from the analysis of ligand rebinding kinetics after photolysis of NP7-CO (presumably at a much lower protein concentration, at which interparticle interactions are negligible). A large fraction of CO ligands rebind geminately in the picosecond time-scale with a biphasic time course. Moreover, nanosecond flash-photolysis experiments clearly show that ligand migration through protein cavities modulates the rebinding process at longer time-scales. Although the main findings are clear already from inspection of the raw data, the procedure used to match the kinetics measured in the sub-nanosecond time-scale with those measured at longer time-scales seems problematic (as also noted by Marten H. Vos in his review of the paper). The detailed procedure used to match the two datasets should be added to the paper. In particular, a supplementary figure showing the two-exponentials fit of the picosecond data and its extrapolation beyond 2 ns, would be helpful in this respect. It would be also useful to have an idea of how much a different choice of the relative scaling between the two datasets would affect the estimation of microscopic rates.

Minor points:

- as noted by Suman Kundu in his review of the paper there are sentences that need to be corrected in the abstract; apart from the second sentence (line 3), also the last sentence (line 17) “Finally, the topological distribution of pockets located around the heme as well as from inner cavities …” clearly needs to be corrected.
- page 3, last paragraph of the Introduction: “Here we report on seven crystal structures of NP7 …”. Only 6 different crystal structures are described in the paper and in Table 1.
- the caption of Figure 12 should be corrected in comparison to Figure 11: T1 should be T2.

We have read this submission. We believe that we have an appropriate level of expertise to confirm that it is of an acceptable scientific standard, however we have significant reservations, as outlined above.

# Peer review: Referee response for version 1

**DOI:** 10.5256/f1000research.6488.r9064

**Published:** 2015-06-23

**Authors:** Suman Kundu

**Affiliations:** 1 Department of Biochemistry, University of Delhi, New Delhi, Delhi, India

Markus Knipp *et al *applied a repertoire of biophysical and computational techniques to provide insight into the unique structural features of NP7, a transporter nitrophorin. The study also highlighted the differences with other nitrophorins and unraveled a likely mechanism for NO migration and exit in NP7, which represents advancement in the understanding of nitrophorins. This is a nice, elaborate piece of work that triggers the mind in many ways.

The article will read better and assume greater significance if the following issues can be addressed:

- The second sentence (line 3) in the Abstract should be modified as “Besides its ability to bind to phospholipid membranes, the N-terminus of NP7, a member of the NO transporter nitrophorin family, contains…………..”;  otherwise the “other” in that sentence does not make any sense.  Moreover, placing NP7 in this sentence also introduces the target protein to the reader better, than mentioning it later. Without such an introduction of the subject of the study, the beginning of the abstract reads pretty abrupt and can be difficult for readers to grasp, unless they come back to it after reading the Introduction.

- How does one ensure that NO stays bound to heme iron in the crystal structures of NPs, given the photoreduction and the fact that Fe(II) might have altered affinity for NO than Fe(III)? The fact that the electron density could not be fit with a single configuration of NO indicates that NO is only partially bound, which means that the crystals contain a mixture of liganded and unliganded states? Does such mixed population influence conclusions about structural features? A sentence in this regard could be added in the relevant section in the manuscript to dispel doubts.  

- Since the authors solved the crystal structures of NP7 at low (5.8) and high pH (7.8) and in NO-bound form, for MD simulations these structures could be used to model the molecular systems rather than using NP4 structures. Alternatively, the models obtained from NP4 could be compared with the crystal structures. Clarification would help readers.   

- Since the liganded and unliganded structures are similar, is it safe to assume that the structure of NP7 is not adapted to regulate ligand binding, but rather acts as a reservoir for NO storage suitably endowed with tunnels and cavities for the NO release as and when required, with dependence on pH only? A line of discussion would help. 

- Finally, as a future perspective, a crystal structure of the mutant of NP7 (with extra N-terminal residues deleted), both in unliganded and liganded forms, might help to validate the rational hypothesis laid down in the paper.

I congratulate the authors on the wonderful manuscript.

I have read this submission. I believe that I have an appropriate level of expertise to confirm that it is of an acceptable scientific standard.

# Peer review: Referee response for version 1

**DOI:** 10.5256/f1000research.6488.r7667

**Published:** 2015-02-24

**Authors:** Marten H. Vos

**Affiliations:** 1 Laboratory of Optics and Biosciences, CNRS, Ecole Polytechnique, Palaiseau, France

This work combines crystallography, time-resolved spectroscopy and MD simulations to investigate structure and CO dynamics in a member of the NO transporter NP family, NP7. Whereas the found properties are similar to other NPs, in particular the well-studied NP4, this work is useful as substantial differences had been suggested from previous studies.

A few suggestions for improvements:

1. Intro, 2 ^nd^ par, 3 ^rd^ phrase suggests that NP1 and NP2 share more identity with NP4 than NP7, which appears not the case for NP2 (Table 2).
2. Please specify sample conditions (path length, concentration) for both types of spectroscopic measurements.
3. MD simulations of NP7 are based on homology modelling from NP4 structures. A comparison of the homology and X-ray models, other than the flipped heme, would be interesting.
4. Admittedly it will not change the order of magnitude of the results, but the problematic gluing of the gap between 1 ns and 20 ns by extrapolating a non-finished double exponential (fit parameters?) can be avoided by determining the effective CO dissociation yield at ~20 ns in the linear excitation regime using an established procedure ( Brunori *et al.*, 1973).
5. If I understand well the kinetics in Fig. 9 can be fitted by four exponentials (two <10 ns and two μs-ms), and thus 7 independent free parameters. The model has as many as 11 rate parameters (Table 3, k _on_ is strictly not a microscopic rate constant). This would suggest the model is underdetermined. Please discuss.
6. Do the authors associate T1, T2 and T3 with specific modeled cavities?
7. It is unclear why the rebinding rate is specifically or only compared with TrHbO. In a number of sensor proteins, including CooA, CO rebinding phases on the order of tens and hundreds of picoseconds have been observed.

I have read this submission. I believe that I have an appropriate level of expertise to confirm that it is of an acceptable scientific standard.
